# Exponential Strong Converse for One Helper Source Coding Problem †

**DOI:** 10.3390/e21060567

**Published:** 2019-06-05

**Authors:** Yasutada Oohama

**Affiliations:** Department of Communication Engineering and Informatics, University of Electro-Communications, Tokyo 182-8585, Japan; oohama@uec.ac.jp; Tel.: +81-42-443-5358

**Keywords:** one helper source coding problem, strong converse theorem, exponent of correct probability of decoding

## Abstract

We consider the one helper source coding problem posed and investigated by Ahlswede, Körner and Wyner. Two correlated sources are separately encoded and are sent to a destination where the decoder wishes to decode one of the two sources with an arbitrary small error probability of decoding. In this system, the error probability of decoding goes to one as the source block length *n* goes to infinity. This implies that we have a strong converse theorem for the one helper source coding problem. In this paper, we provide the much stronger version of this strong converse theorem for the one helper source coding problem. We prove that the error probability of decoding tends to one exponentially and derive an explicit lower bound of this exponent function.

## 1. Introduction

For single or multi terminal source encoding systems, the converse coding theorems state that, at any data compression rates below the fundamental theoretical limit of the system, the error probability of decoding *can not go to zero* when the block length *n* of the codes tends to infinity.

**Figure 1:**
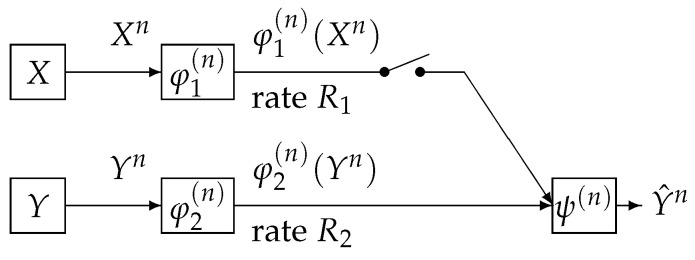
Source encoding with or without side information at the decoder.

In this paper, we study the one helper source coding problem posed and investigated by Ahlswede, Körner [1] and Wyner [2]. We call the above source coding system (the AKW system). The AKW system is shown in Figure 1.

In this figure, the AKW system corresponds to the case where the switch is closed. In Figure 1, the sequence $\left(X^{n},Y^{n}\right)$ represents independent copies of a pair of dependent random variables (X,Y) which take values in the finite sets X,Y, respectively. We assume that (X,Y) has a probability distribution denoted by $p_{XY}$. For each i=1,2, the encoder $\varphi_{i}^{\left(n\right)}$ outputs a binary sequence which appears at a rate $R_{i}$ bits per input symbol. The decoder function $\psi^{\left(n\right)}$ observes $\varphi_{1}^{\left(n\right)}\left(X^{n}\right)$ and $\varphi_{2}^{\left(n\right)}\left(Y^{n}\right)$ to output a sequence ${\hat{Y}}^{n}:=\psi^{\left(n\right)}\left(\varphi_{1}^{\left(n\right)}\left(X^{n}\right),\varphi_{2}^{\left(n\right)}\left(Y^{n}\right)\right)$, which is an estimation of $Y^{n}$. When the switch is open, it is well known that the minimum transmission rate $R_{2}$ such that the error probability $P_{e}^{\left(n\right)}:=\mathrm{Pr}\left\{Y^{n}\neq {\hat{Y}}^{n}\right\}$ of decoding tends to zero as *n* tends to infinity is given by H(Y). Csiszár and Longo [3] proved that, if $R_{2}<H\left(Y\right)$, then the correct probability $P_{c}^{\left(n\right)}:=\mathrm{Pr}\left\{Y^{n}={\hat{Y}}^{n}\right\}$ of decoding decay exponentially and derived the optimal exponent function. When the switch is open and $R_{1}>H\left(X\right)$, Slepian and Wolf [4] proved that H(Y|X) is the minimum transmission rate $R_{2}$ such that the error probability $\mathrm{Pr}\{Y^{n}\neq {\hat{Y}}^{n}\}$ of decoding tends to zero as *n* tends to infinity. Oohama and Han [5] proved that, if $R_{2}<H\left(Y|X\right)$, then the correct probability $P_{c}^{\left(n\right)}:=\mathrm{Pr}\left\{Y^{n}={\hat{Y}}^{n}\right\}$ of decoding decay exponentially and derived the optimal exponent function.

In this paper, we consider the strong converse theorem in the case where the switch is closed and $0<R_{1}<H\left(X\right)$. Let $R_{\mathrm{AKW}}\left(p_{XY}\right)$ be the rate region of the AKW system. This region consists of the rate pair $\left(R_{1},R_{2}\right)$ such that the error provability of decoding goes to zero as *n* tends to infinity. The rate region was determined by Ahlswede, Körner [1] and Wyner [2]. On the converse coding theorem, Ahlswede et al. [6] proved that, if $\left(R_{1},R_{2}\right)$ is outside the rate region, then, $P_{c}^{\left(n\right)}$ must tends to zero as *n* tends to infinity. Gu and Effors [7] examined a speed of convergence for $P_{c}^{\left(n\right)}$ to tend to zero as n→∞ by carefully checking the proof of Ahlswede et al. [6]. However, they could not obtain a result on an explicit form of the exponent function with respect to the code length *n*.

Our main results on the strong converse theorem for the AKW system are as follows. For the AKW system, we prove that, if $\left(R_{1},R_{2}\right)$ is outside the rate region $R_{\mathrm{AKW}}\left(p_{XY}\right)$, $P_{c}^{\left(n\right)}$ must go to zero exponentially and derive an explicit lower bound of this exponent. This result corresponds to Theorem 3. As a corollary from this theorem, we obtain the strong converse result, which is stated in Corollary 2. This result states that we have an outer bound with $O(1/\sqrt{n})$ gap from the rate region $R_{\mathrm{AKW}}\left(p_{XY}\right)$.

To derive our result, we use a new method called the recursive method. This method, which is a new method introduced by the author, includes a certain recursive algorithm for a single letterization of exponent functions. In a standard argument of proving converse coding theorems, single letterization methods based on the chain rule of the entropy functions are used. In general, the functions representing multi letter characterizations of exponent functions do not have the chain rule property. In such cases, the recursive method is quite useful for deriving single letterized bounds. The recursive method is a general powerful tool to prove strong converse theorems for several coding problems in information theory. In fact, the recursive method plays important roles in deriving exponential strong converse exponent for communication systems treated in [8,9,10,11,12].

On the strong converse theorem for the one helper source coding problem, we have two recent other works [13,14]. The above two works proved the strong converse theorem using different methods from our method. In [13], Watanabe found a relationship between the AKW system and the Gray–Wyner network. Using this relationship and the second order rate region for the Gray–Wyner network obtained by him [15], Watanabe established the strong converse theorem for the AKW system. In [14], Liu et al. introduced a new method to derive sharp strong converse bounds via a reverse hypercontractivity. Using this method, they obtained an outer bound of the rate region for the AKW system with $O(1/\sqrt{n})$ gap from the rate region. Furthermore, in [14], an extension of the AKW system to the case of Gaussian source and quadratic distortion is investigated, obtaining an outer bound with $O(1/\sqrt{n})$ gap from the rate distortion region for the extended source coding system. In his resent paper [16], Liu showed a lower bound (converse) on the dispersion of AWK as the variance of the linear combination of information densities.

The strong converse theorems seem to be regarded just as a mathematical problem and have been investigated mainly from theoretical interest. Recently, Watanabe and Oohama [17] have found an interesting security problem, which has a close connection with the strong converse theorem for the AKW system. Furthermore, Oohama and Santoso [18] and Santoso and Oohama [19] clarify that the exponential strong converse theorem obtained by this paper plays an essential role in deriving a strong sufficient secure condition for the privacy amplification in their new theoritical model of side channel attacks to the Shannon chipher systems. From the above two cases, we expect that exponential strong converse theorems for multiterminal source networks will serve as a strong tool to several information theoretical security problems.

## 2. Problem Formulation

Let X and Y be finite sets and ${\left\{(X_{t},Y_{t})\right\}}_{t=1}^{\infty }$ be a stationary discrete memoryless source. For each t=1,2,⋯, the random pair $\left(X_{t},Y_{t}\right)$ takes values in X×Y, and has a probability distribution

$$p_{XY}={\left\{p_{XY}\left(x,y\right)\right\}}_{(x,y)\in X\times Y}.$$

We write *n* independent copies of ${\left\{X_{t}\right\}}_{t=1}^{\infty }$ and ${\left\{Y_{t}\right\}}_{t=1}^{\infty }$, respectively as

$$X^{n}=X_{1},X_{2},\cdots ,X_{n}\;\mathrm{and}\;Y^{n}=Y_{1},Y_{2},\cdots ,Y_{n}.$$

**Figure 2:**
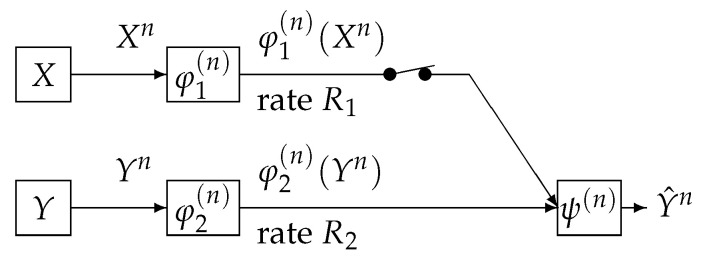
One helper source coding system [20].

We consider a communication system depicted in Figure 2. This communication system corresponds to the case where the switch is closed in Figure 1. Data sequences $X^{n}$ and $Y^{n}$ are separately encoded to $\varphi_{1}^{\left(n\right)}\left(X^{n}\right)$ and $\varphi_{2}^{\left(n\right)}\left(Y^{n}\right)$ and those are sent to the information processing center. At the center, the decoder function $\psi^{\left(n\right)}$ observes $\left(\varphi_{1}^{\left(n\right)}\left(X^{n}\right),\varphi_{2}^{\left(n\right)}\left(Y^{n}\right)\right)$ to output the estimation ${\hat{Y}}^{n}$ of $Y^{n}$. The encoder functions $\varphi_{1}^{\left(n\right)}$ and $\varphi_{2}^{\left(n\right)}$ are defined by

(1) $$\left.\begin{array}{c} \varphi_{1}^{\left(n\right)}:X^{n}\to M_{1}=\left\{\;1,2,\cdots ,M_{1}\;\right\}\;\\ \varphi_{2}^{\left(n\right)}:Y^{n}\to M_{2}=\left\{\;1,2,\cdots ,M_{2}\;\right\} \end{array}\right\},$$

where for each i=1,2, $∥\varphi_{i}^{\left(n\right)}∥$$\left(=M_{i}\right)$ stands for the range of cardinality of $\varphi_{i}^{\left(n\right)}$. The decoder function $\psi^{\left(n\right)}$ is defined by

(2) $$\psi^{\left(n\right)}:M_{1}\times M_{2}\;\to \;Y^{n}.$$

The error probability of decoding is

(3) $$P_{e}^{\left(n\right)}\left(\varphi_{1}^{\left(n\right)},\varphi_{2}^{\left(n\right)},\psi^{\left(n\right)}\right)=\mathrm{Pr}\left\{{\hat{Y}}^{n}\neq Y^{n}\right\},$$

where ${\hat{Y}}^{n}=\psi^{\left(n\right)}\left(\varphi_{1}^{\left(n\right)}\left(X^{n}\right),\varphi_{2}^{\left(n\right)}\left(Y^{n}\right)\right)$. A rate pair $\left(R_{1},R_{2}\right)$ is ε-*achievable* if, for any δ>0, there exists a positive integer $n_{0}=n_{0}\left(\varepsilon ,\delta \right)$ and a sequence of triples $\{(\varphi_{1}^{\left(n\right)},$$\varphi_{2}^{\left(n\right)},$ $\psi^{\left(n\right)}{)\}}_{n\geq n_{0}}$ such that, for $n\geq n_{0}$,

$$\begin{array}{c} \frac{1}{n}\log∥\varphi_{i}^{\left(n\right)}∥\leq R_{i}+\delta \;\mathrm{for}\;i=1,2,P_{e}^{\left(n\right)}\left(\varphi_{1}^{\left(n\right)},\varphi_{2}^{\left(n\right)},\psi^{\left(n\right)}\right)\leq \varepsilon . \end{array}$$

For ε∈(0,1), the rate region $R_{\mathrm{AKW}}\left(\varepsilon |p_{XY}\right)$ is defined by

$$\begin{array}{c} R_{\mathrm{AKW}}\left(\varepsilon |p_{XY}\right):=\left\{\;\left(R_{1},R_{2}\right):\left(R_{1},R_{2}\right)\;\mathrm{is}\varepsilon -\mathrm{achievable}\;\mathrm{for}p_{XY}\;\right\}. \end{array}$$

Furthermore, define

$$R_{\mathrm{AKW}}\left(p_{XY}\right):=\underset{\varepsilon \in (0,1)}{⋂}R_{\mathrm{AKW}}\left(\varepsilon |p_{XY}\right).$$

We can show that the two rate regions $R_{\mathrm{AKW}}\left(\varepsilon \right|$$p_{XY})$, ε∈(0,1) and $R_{\mathrm{AKW}}\left(p_{XY}\right)$ satisfy the following property.

**Property** **1.**

*(a)*  *The regions $R_{\mathrm{AKW}}\left(\varepsilon |p_{XY}\right)$, ε∈(0,1), and $R_{\mathrm{AKW}}($$p_{XY})$ are closed convex sets of $R_{+}^{2}$, where*
  $$\begin{array}{cc} R_{+}^{2} & :=\{\left(R_{1},R_{2}\right):R_{1}\geq 0,R_{2}\geq 0\}. \end{array}$$
*(b)*  *$R_{\mathrm{AKW}}\left(\varepsilon |p_{XY}\right)$ has another form using (n,ε)-rate region $R_{\mathrm{AKW}}\left(n,\varepsilon |p_{XY}\right)$, the definition of which is as follows. We set*
  $$\begin{array}{c} R_{\mathrm{AKW}}\left(n,\varepsilon |p_{XY}\right)=\{\left(R_{1},R_{2}\right):There\;exists\left(\varphi_{1}^{\left(n\right)},\varphi_{2}^{\left(n\right)},\psi^{\left(n\right)}\right)such\;that\\ \frac{1}{n}\log||\varphi_{i}^{\left(n\right)}\left|\right|\leq R_{i},i=1,2,P_{e}^{\left(n\right)}\left(\varphi_{1}^{\left(n\right)},\varphi_{2}^{\left(n\right)},\psi^{\left(n\right)}\right)\leq \varepsilon \}. \end{array}$$
*Using $R_{\mathrm{AKW}}(n,$$\varepsilon |p_{XY})$, $R_{\mathrm{AKW}}\left(\varepsilon |p_{XY}\right)$ can be expressed as*
  $$\begin{array}{cc} R_{\mathrm{AKW}}\left(\varepsilon |p_{XY}\right) & =\mathrm{cl}\left(\underset{m\geq 1}{⋃}\underset{n\geq m}{⋂}R_{\mathrm{AKW}}\left(n,\varepsilon |p_{XY}\right)\right). \end{array}$$

Proof of this property is given in Appendix A. It is well known that $R_{\mathrm{AKW}}\left(p_{XY}\right)$ was determined by Ahlswede, Körner and Wyner. To describe their result, we introduce an auxiliary random variable *U* taking values in a finite set U. We assume that the joint distribution of (U,X,Y) is

$$p_{UXY}\left(u,x,y\right)=p_{U}\left(u\right)p_{X|U}\left(x|u\right)p_{Y|X}\left(y|x\right).$$

The above condition is equivalent to U↔X↔Y. Define the set of probability distribution $p=p_{UXY}$ by

$$\begin{array}{c} P\left(p_{XY}\right):=\{p_{UXY}:|U|\leq |X|+1,U↔X↔Y\}. \end{array}$$

Set

$$\begin{array}{cc} R(p) & :=\begin{array}{c} \left\{ \left(R_{1},R_{2}\right):R_{1},R_{2}\geq 0\;\begin{array}{ccc} R_{1} & \geq & I_{p}\left(X;U\right),R_{2}\geq H_{p}\left(Y|U\right)\}, \end{array}\right. \end{array}\\ R(p_{XY}) & :=\underset{p\in P(p_{XY})}{⋃}R\left(p\right). \end{array}$$

We can show that the region $R(p_{XY})$ satisfies the following property.

**Property** **2.**

*(a)*  *The region $R(p_{XY})$ is a closed convex subset of $R_{+}^{2}$.*
*(b)*  *For any $p_{XY}$, we have*
  (4) $$\min_{\left(R_{1},R_{2}\right)\in R\left(p_{XY}\right)}\left(R_{1}+R_{2}\right)=H_{p}\left(Y\right).$$
*The minimum is attained by $\left(R_{1},R_{2}\right)=\left(0,H_{p}\left(Y\right)\right)$. This result implies that*
  $$\begin{array}{c} R\left(p_{XY}\right)\subseteq \left\{\left(R_{1},R_{2}\right):R_{1}+R_{2}\geq H_{p}\left(Y\right)\right\}\cap R_{+}^{2}. \end{array}$$
*Furthermore, the point $\left(0,H_{p}\left(Y\right)\right)$ always belongs to $R(p_{XY})$.*

**Figure 3:**
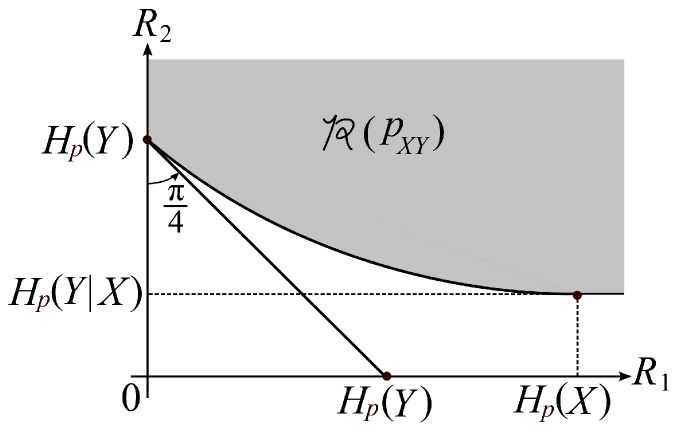
A typical shape of $R(p_{XY})$.

Property 2 part a is a well known property. Proof of Property 2 part b is easy. Proofs of Property 2 parts a and b are omitted. A typical shape of the rate region $R(p_{XY})$ is shown in Figure 3.

The rate region $R_{\mathrm{AKW}}\left(p_{XY}\right)$ was determined by Ahlswede and Körner [1] and Wyner [2]. Their results are the following.

**Theorem** **1** (Ahlswede, Körner [1] and Wyner [2]).

$$\begin{array}{c} R_{\mathrm{AKW}}\left(p_{XY}\right)=R\left(p_{XY}\right). \end{array}$$

On the converse coding theorem, Ahlswede et al. [6] obtained the following.

**Theorem** **2** (Ahlswede et al. [6]). *For each fixed ε∈(0,1), we have*

$$\begin{array}{c} R_{\mathrm{AKW}}\left(\varepsilon |p_{XY}\right)=R\left(p_{XY}\right). \end{array}$$

Gu and Effors [7] examined a speed of convergence for $P_{e}^{\left(n\right)}$ to tend to 1 as n→∞ by carefully checking the proof of Ahlswede et al. [6]. However, they could not obtain a result on an explicit form of the exponent function with respect to the code length *n*.

Our aim is to find an explicit form of the exponent function for the error probability of decoding to tend to one as n→∞ when $\left(R_{1},R_{2}\right)\notin R_{\mathrm{AKW}}\left(p_{XY}\right)$. To examine this quantity, we define the following quantity. Set

Pc(n)(φ1(n),φ2(n),ψ(n)):=1−Pe(n)(φ1(n),φ2(n),ψ(n)),G(n)(R1,R2|pXY):=min(φ1(n),φ2(n),ψ(n)):(1/n)log∥φi(n)∥≤Ri,i=1,2−1nlogPc(n)(φ1(n),φ2(n),ψ(n)).G(R1,R2|pXY):=limn→∞G(n)(R1,R2|pXY),G(pXY):={(R1,R2,G):G≥G(R1,R2|pXY)}.

By time sharing, we have that

(5) $$\begin{array}{c} G^{\left(n+m\right)}\left(\left.\frac{nR_{1}+mR_{1}^{\prime }}{n+m},\frac{nR_{2}+mR_{2}^{\prime }}{n+m}\right|p_{XY}\right)\leq \frac{nG^{\left(n\right)}\left(R_{1},R_{2}|p_{XY}\right)+mG^{\left(m\right)}\left(R_{1}^{\prime },R_{2}^{\prime }|p_{XY}\right)}{n+m}. \end{array}$$

Choosing $R=R^{\prime }$ in the inequality (5), we obtain the following subadditivity property on $\{G^{\left(n\right)}\left(R_{1},R_{2}|p_{XY}\right)$${\}}_{n\geq 1}$:

$$\begin{array}{c} G^{\left(n+m\right)}\left(R_{1},R_{2}|p_{XY}\right)\leq \frac{nG^{\left(n\right)}\left(R_{1},R_{2}|p_{XY}\right)+mG^{\left(m\right)}\left(R_{1},R_{2}|p_{XY}\right)}{n+m}, \end{array}$$

from which this, and Fekete’s subadditive lemma, we have that $G^{\left(n\right)}\left(R_{1},R_{2}|p_{XY}\right)$ exists and satisfies the following:

$$\begin{array}{c} \lim_{n\to \infty }G^{\left(n\right)}\left(R_{1},R_{2}|p_{XY}\right)=\underset{n\geq 1}{\inf}G^{\left(n\right)}\left(R_{1},R_{2}|p_{XY}\right). \end{array}$$

The exponent function $G(R_{1},R_{2}|p_{XY})$ is a convex function of $\left(R_{1},R_{2}\right)$. In fact, from the inequality (5), we have that for any α∈[0,1]

$$\begin{array}{c} G\left(\alpha R_{1}+\bar{\alpha }R_{1}^{\prime },\alpha R_{2}+\bar{\alpha }R_{2}^{\prime }|p_{XY}\right)\leq \alpha G\left(R_{1},R_{2}|p_{XY}\right)+\bar{\alpha }G\left(R_{1}^{\prime },R_{2}^{\prime }|p_{XY}\right). \end{array}$$

The region $G(p_{XY})$ is also a closed convex set. Our main aim is to find an explicit characterization of $G(p_{XY})$. In this paper, we derive an explicit outer bound of G$\left(p_{XY}\right)$ whose section by the plane G=0 coincides with $R_{\mathrm{AKW}}\left(p_{XY}\right)$.

## 3. Main Results

In this section, we state our main result. We first explain that the region $R(p_{XY})$ can be expressed with a family of supporting hyperplanes. To describe this result, we define a set of probability distributions on U×X×Y by

$$\begin{array}{cc} P_{\mathrm{sh}}\left(p_{XY}\right) & :=\{p=p_{UXY}:|U|\leq |X|,U↔X↔Y\}. \end{array}$$

For μ≥0, define

$$\begin{array}{c} R^{\left(\mu \right)}\left(p_{XY}\right):=\min_{p\in P_{\mathrm{sh}}\left(p_{XY}\right)}\left\{\mu I_{p}\left(X;U\right)+\bar{\mu }H_{p}\left(Y|U\right)\right\}. \end{array}$$

Furthermore, define

$$\begin{array}{c} R_{\mathrm{sh}}\left(p_{XY}\right):=\underset{\mu \in [0,1]}{⋂}\left\{(R_{1},R_{2}\right):\begin{array}{c} \mu R_{1}+\bar{\mu }R_{2}\geq R^{\left(\mu \right)}\left(p_{XY}\right)\}. \end{array} \end{array}$$

Then, we have the following property.

**Property** **3.**

*(a)*  *The bound |U|≤|X| is sufficient to describe $R^{\left(\mu \right)}($$p_{XY})$.*
*(b)*  *For every μ∈[0,1], we have*
  (6) $$\begin{array}{c} \min_{\left(R_{1},R_{2}\right)\in R\left(p_{XY}\right)}\left\{\mu R_{1}+\bar{\mu }R_{2}\right\}=R^{\left(\mu \right)}\left(p_{XY}\right). \end{array}$$
*(c)*  *For any $p_{XY},$ we have*
  (7) $$R_{\mathrm{sh}}\left(p_{XY}\right)=R\left(p_{XY}\right).$$

Property 3 part a is stated as Lemma A1 in Appendix B. Proof of this lemma is given in this appendix. Proofs of Property 3 parts b and c are given in Appendix C. Set

$$\begin{array}{cc} Q(p_{Y|X}) & :=\{q=q_{UXY}:|U|\leq |X|,U↔X↔Y,p_{Y|X}=q_{Y|X}\}. \end{array}$$

For $\left(\mu ,\alpha \right)\in {\left[0,1\right]}^{2}$, and for $q=q_{UXY}\in Q\left(p_{Y|X}\right)$, define

ωq|pX(μ,α)(x,y|u):=α¯logqX(x)pX(x)+αμlogqX|U(x|u)pX(x)+μ¯log1qY|U(y|u),fq|pX(μ,α)(x,y|u):=exp−ωq|pX(μ,α)(x,y|u),Ω(μ,α)(q|pX):=−logEqexp−ωq|pX(μ,α)(X,Y|U),Ω(μ,α)(pXY):=minq∈Q(pY|X)Ω(μ,α)(q|pX),F(μ,α)(μR1+μ¯R2|pXY):=Ω(μ,α)(pXY)−α(μR1+μ¯R2)2+αμ¯,F(R1,R2|pXY):=sup(μ,α)∈[0,1]2F(μ,α)(μR1+μ¯R2|pXY).

We next define a function serving as a lower bound of $F(R_{1},R_{2}|p_{XY})$. For λ≥0 and for $p_{UXY}\in P_{\mathrm{sh}}\left(p_{XY}\right)$, define

ω˜p(μ)(x,y|u):=μlogpX|U(x|u)pX(x)+μ¯log1pY|U(y|u),Ω˜(μ,λ)(p):=−logEpexp−λω˜p(μ)(X,Y|U),Ω˜(μ,λ)(pXY):=minp∈Psh(pXY)Ω˜(μ,λ)(p).

Furthermore, set

$$\begin{array}{c} {\underline{F}}^{\left(\mu ,\lambda \right)}\left(\mu R_{1}+\bar{\mu }R_{2}|p_{XY}\right):=\frac{{\tilde{\Omega }}^{\left(\mu ,\lambda \right)}\left(p_{XY}\right)-\lambda \left(\mu R_{1}+\bar{\mu }R_{2}\right)}{2+\lambda (5-\mu )},\\ \underline{F}\left(R_{1},R_{2}|p_{XY}\right):=\underset{\lambda \geq 0,\mu \in [0,1]}{\sup}{\underline{F}}^{\left(\mu ,\lambda \right)}\left(\mu R_{1}+\bar{\mu }R_{2}|p_{XY}\right). \end{array}$$

We can show that the above functions satisfy the following property.

**Property** **4.**

*(a)*  *The cardinality bound |U|≤|X| in $Q(p_{Y|X})$ is sufficient to describe the quantity $\Omega^{\left(\mu ,\alpha \right)}\left(p_{XY}\right)$. Furthermore, the cardinality bound |U|≤|X| in $P_{\mathrm{sh}}\left(p_{XY}\right)$ is sufficient to describe the quantity ${\tilde{\Omega }}^{\left(\mu ,\lambda \right)}\left(p_{XY}\right)$.*
*(b)*  *For any $R_{1},R_{2}\geq 0$, we have*
  $$\begin{array}{c} F\left(R_{1},R_{2}|p_{XY}\right)\geq \underline{F}\left(R_{1},R_{2}|p_{XY}\right). \end{array}$$
*(c)*  *For any $p=p_{UXY}\in P_{\mathrm{sh}}\left(p_{XY}\right)$ and any (μ,λ${)\in \left[0,1\right]}^{2}$, we have*
  (8) $$0\leq {\tilde{\Omega }}^{\left(\mu ,\lambda \right)}\left(p\right)\leq \mu \log|X|+\bar{\mu }\log\left|Y\right|.$$
*(d)*  *Fix any $p=p_{UXY}\in P_{\mathrm{sh}}\left(p_{XY}\right)$ and μ∈[0,1]. For λ∈[0,1], we define a probability distribution $p^{\left(\lambda \right)}=p_{UXY}^{\left(\lambda \right)}$ by*
  $$\begin{array}{c} p^{\left(\lambda \right)}\left(u,x,y\right):=\frac{p\left(u,x,y\right)\exp\left\{-\lambda {\tilde{\omega }}_{p}^{\left(\mu \right)}\left(x,y|u\right)\right\}}{E_{p}\left[\exp\left\{-\lambda {\tilde{\omega }}_{p}^{\left(\mu \right)}\left(X,Y|U\right)\right\}\right]}. \end{array}$$
*Then, for λ∈[0,1/2], ${\tilde{\Omega }}^{\left(\mu ,\lambda \right)}\left(p\right)$ is twice differentiable. Furthermore, for λ∈[0,1/2], we have*
  $$\begin{array}{c} \frac{d}{d\lambda }{\tilde{\Omega }}^{\left(\mu ,\lambda \right)}\left(p\right)=E_{p^{\left(\lambda \right)}}\left[{\tilde{\omega }}_{p}^{\left(\mu \right)}\left(X,Y|U\right)\right],\frac{d^{2}}{d\lambda^{2}}{\tilde{\Omega }}^{\left(\mu ,\lambda \right)}\left(p\right)=-{\mathrm{Var}}_{p^{\left(\lambda \right)}}\left[{\tilde{\omega }}_{p}^{\left(\mu \right)}\left(X,Y|U\right)\right]. \end{array}$$
*The second equality implies that ${\tilde{\Omega }}^{\left(\mu ,\lambda \right)}(p$$|p_{XY})$ is a concave function of λ∈[0,1/2].*
*(e)*  *For every (μ,λ)∈[0,1]×[0,1/2], define*
  ρ(μ,λ)(pXY):=max(ν,p)∈[0,λ]×Psh(pXY):Ω˜(μ,λ)(p)=Ω˜(μ,λ)(pXY)Varp(ν)ω˜p(μ)(X,Y|U),
  *and set*
  $$\begin{array}{c} \rho =\rho \left(p_{XY}\right):=\max_{\left(\mu ,\lambda )\in [0,1]\times [0,1/2\right]}\rho^{\left(\mu ,\lambda \right)}\left(p_{XY}\right). \end{array}$$
*Then, we have $\rho (p_{XY})<\infty .$ Furthermore, for any (μ,λ)∈[0,1]×[0,1/2], we have*
  (9) $${\tilde{\Omega }}^{\left(\mu ,\lambda \right)}\left(p_{XY}\right)\geq \lambda R^{\left(\mu \right)}\left(p_{XY}\right)-\frac{\lambda^{2}}{2}\rho \left(p_{XY}\right).$$
*(f)*  *For every $\tau \in (0,\left(1/2\right)\rho \left(p_{XY}\right))$, the condition $(R_{1}+\tau ,$$R_{2}+\tau )\notin R\left(p_{XY}\right)$ implies*
  $$\underline{F}\left(R_{1},R_{2}|p_{XY}\right)>\frac{\rho (p_{XY})}{4}\cdot g^{2}\left(\frac{\tau }{\rho (p_{XY})}\right)>0,$$
  *where g is the inverse function of $\vartheta \left(a\right):=a+\left(5/4\right)a^{2},a\geq 0$.*

Property 3 part a is stated as Lemma A2 in Appendix B. Proof of this lemma is given in this appendix. Proof of Property 4 part b is given in Appendix D. Proofs of Property 4 parts c, d, e, and f are given in Appendix E.

Our main result is the following.

**Theorem** **3.** *For any $R_{1},R_{2}\geq 0$, any $p_{XY}$, and for any $(\varphi_{1}^{\left(n\right)},$$\varphi_{1}^{\left(n\right)},$$\psi^{\left(n\right)})$ satisfying $\left(1/n\right)\log||\varphi_{i}^{\left(n\right)}\left|\right|\leq R_{i},i=1,2,$ we have*

(10) $$P_{c}^{\left(n\right)}\left(\varphi_{1}^{\left(n\right)},\varphi_{2}^{\left(n\right)},\psi^{\left(n\right)}\right)\leq 5\exp\left\{-nF(R_{1},R_{2}|p_{XY})\right\}.$$

It can be seen from Property 4 parts b and f that $F(R_{1},R_{2}|p_{XY})$ is strictly positive if $\left(R_{1},R_{2}\right)$ is outside the rate region $R(p_{XY})$. Hence, by Theorem 3, we have that, if $\left(R_{1},R_{2}\right)$ is outside the rate region, then the error probability of decoding goes to one exponentially and its exponent is not below $F(R_{1},R_{2}|p_{XY})$. It immediately follows from Theorem 3 that we have the following corollary.

**Corollary** **1.** $$\begin{array}{c} G\left(R_{1},R_{2}|p_{XY}\right)\geq F\left(R_{1},R_{2}|p_{XY}\right),\\ G\left(p_{XY}\right)\subseteq \bar{G}\left(p_{XY}\right)=\left\{\left(R_{1},R_{2},G\right):G\geq F\left(R_{1},R_{2}|p_{XY}\right)\right\}. \end{array}$$

Proof of Theorem 3 will be given in the next section. The exponent function at rates outside the rate region was derived by Oohama and Han [5] for the separate source coding problem for correlated sources [4]. The techniques used by them is a method of types [21], which is not useful to prove Theorem 3. Some novel techniques based on the information spectrum method introduced by Han [22] are necessary to prove this theorem.

From Theorem 3 and Property 4 part e, we can obtain an explicit outer bound of $R_{\mathrm{AKW}}\left(\varepsilon |p_{XY}\right)$ with an asymptotically vanishing deviation from $R_{\mathrm{AKW}}\left(p_{XY}\right)$$=R(p_{XY})$. The strong converse theorem established by Ahlswede et al. [6] immediately follows from this corollary. To describe this outer bound, for κ>0, we set

$$R\left(p_{XY}\right)-\kappa \left(1,1\right):=\left\{\left(R_{1}-\kappa ,R_{2}-\kappa \right):\left(R_{1},R_{2}\right)\in R\left(p_{XY}\right)\right\},$$

which serves as an outer bound of $R(p_{XY})$. For each fixed ε∈(0,1), we define $\kappa_{n}$$=\kappa_{n}\left(\varepsilon ,\rho \left(p_{XY}\right)\right)$ by

(11) $$\begin{array}{ccc} \kappa_{n} & := & \rho \left(p_{XY}\right)\vartheta \left(\sqrt{\frac{4}{n\rho (p_{XY})}\log\left(\frac{5}{1-\varepsilon }\right)}\right)\\ & \overset{\left(a\right)}{=} & 2\sqrt{\frac{\rho (p_{XY})}{n}\log\left(\frac{5}{1-\varepsilon }\right)}+\frac{5}{n}\log\left(\frac{5}{1-\varepsilon }\right). \end{array}$$

Step (a) follows from $\vartheta \left(a\right)=a+\left(5/4\right)a^{2}$. Since $\kappa_{n}\to 0$ as n→∞, we have the smallest positive integer $n_{0}=n_{0}\left(\varepsilon ,\rho \left(p_{XY}\right)\right)$ such that $\kappa_{n}\leq \left(1/2\right)\rho \left(p_{XY}\right)$ for $n\geq n_{0}$. From Theorem 3 and Property 4 part e, we have the following corollary.

**Corollary** **2.** *For each fixed ε∈(0,1), we choose the above positive integer $n_{0}=$$n_{0}\left(\varepsilon ,\rho \left(p_{XY}\right)\right)$. Then, for any $n\geq n_{0}$, we have*

$$\begin{array}{c} R_{\mathrm{AKW}}\left(n,\varepsilon |p_{XY}\right)\subseteq R\left(p_{XY}\right)-\kappa_{n}\left(1,1\right). \end{array}$$

*The above result together with*

$$\begin{array}{cc} R_{\mathrm{AKW}}\left(\varepsilon |p_{XY}\right) & =\mathrm{cl}\left(\underset{m\geq 1}{⋃}\underset{n\geq m}{⋂}R_{\mathrm{AKW}}\left(n,\varepsilon |p_{XY}\right)\right) \end{array}$$

*yields that, for each fixed ε∈(0,1), we have*

$$\begin{array}{c} R_{\mathrm{AKW}}\left(\varepsilon |p_{XY}\right)=R_{\mathrm{AKW}}\left(p_{XY}\right)=R\left(p_{XY}\right). \end{array}$$

*This recovers the strong converse theorem proved by Ahlswede et al. [6].*

Proof of this corollary will be given in the next section.

## 4. Proof of the Main Result

Let $\left(X^{n},Y^{n}\right)$ be a pair of random variables from the information source. We set $S=\varphi_{1}^{\left(n\right)}\left(X^{n}\right)$. Joint distribution $p_{SX^{n}Y^{n}}$ of $\left(S,X^{n},Y^{n}\right)$ is given by

$$\begin{array}{c} p_{SX^{n}Y^{n}}\left(s,x^{n},y^{n}\right)=p_{S|X^{n}}\left(s|x^{n}\right)\prod_{t=1}^{n}p_{X_{t}Y_{t}}\left(x_{t},y_{t}\right). \end{array}$$

It is obvious that $S↔X^{n}↔Y^{n}$. Then, we have the following lemma, which is well known as a single shot infomation spectrum bound.

**Lemma** **1.** *For any η>0 and for any $(\varphi_{1}^{\left(n\right)}$, $\varphi_{2}^{\left(n\right)},\psi^{\left(n\right)})$ satisfying $\left(1/n\right)\log\left|\right|\varphi_{i}^{\left(n\right)}\left|\right|\leq R_{i},i=1,2,$ we have*

(12) $$\begin{array}{cc} P_{c}^{\left(n\right)}\left(\varphi_{1}^{\left(n\right)},\varphi_{2}^{\left(n\right)},\psi^{\left(n\right)}\right)\leq p_{SX^{n}Y^{n}}\{ & \;\;\;0\geq \frac{1}{n}\log\frac{{\hat{q}}_{{SX^{n}Y}^{n}}\left(S,X^{n},Y^{n}\right)}{p_{SX^{n}Y^{n}}\left(S,X^{n},Y^{n}\right)}-\eta , \end{array}$$

(13) $$\begin{array}{c} \;\;\;0\geq \frac{1}{n}\log\frac{Q_{X^{n}}\left(X^{n}\right)}{p_{X^{n}}\left(X^{n}\right)}-\eta , \end{array}$$

(14) $$\begin{array}{c} R_{1}\geq \frac{1}{n}\log\frac{{\tilde{Q}}_{X^{n}|S}\left(X^{n}|S\right)}{p_{X^{n}}\left(X^{n}\right)}-\eta , \end{array}$$

(15) $$\begin{array}{c} R_{2}\geq \left.\frac{1}{n}\log\frac{1}{p_{Y^{n}|S}\left(Y^{n}|S\right)}-\eta \right\}+4e^{-n\eta }. \end{array}$$

*The probability distributions appearing in the three inequalities (12), (13), and (14) in the right members of (15) have a property that we can select them as arbitrary. In (12), we can choose any probability distribution ${\hat{q}}_{SX^{n}Y^{n}}$ on S$\times X^{n}$$\times Y^{n}$. In (13), we can choose any distribution $Q_{X^{n}}$ on $X^{n}$. In (14), we can choose any stochastic matrix ${\tilde{Q}}_{X^{n}|U^{n}}$: $X^{n}$$\to U^{n}$.*

This lemma can be proved by a standard argument in the information spectrum method [22]. The detail of the proof is given in Appendix F. Next, we single letterize the four information spectrum quantities inside the first term in the right members of (15) in Lemma 1 to obtain the following lemma.

**Lemma** **2.** *For any η>0 and for any $(\varphi_{1}^{\left(n\right)}$, $\varphi_{2}^{\left(n\right)},\psi^{\left(n\right)})$ satisfying $\left(1/n\right)\log\left|\right|\varphi_{i}^{\left(n\right)}\left|\right|\leq R_{i},i=1,2,$ we have*

(16) $$\begin{array}{c} P_{c}^{\left(n\right)}\left(\varphi_{1}^{\left(n\right)},\varphi_{2}^{\left(n\right)},\psi^{\left(n\right)}\right)\leq p_{SX^{n}Y^{n}}\left\{0\geq \frac{1}{n}\sum_{t=1}^{n}\log\frac{Q_{X_{t}}\left(X_{t}\right)}{p_{X_{t}}\left(X_{t}\right)}-\eta ,\right. \end{array}$$

(17) $$\begin{array}{c} R_{1}\geq \frac{1}{n}\sum_{t=1}^{n}\log\frac{{\tilde{Q}}_{X_{t}|SX^{t-1}}\left(X_{t}|S,X^{t-1}\right)}{p_{X_{t}}\left(X_{t}\right)}-\eta , \end{array}$$

$$\begin{array}{c} R_{2}\geq \left.\frac{1}{n}\sum_{t=1}^{n}\log\frac{1}{p_{Y_{t}|SX^{t-1}Y^{t-1}}\left(Y_{t}|S,X^{t-1},Y^{t-1}\right)}-2\eta \right\}+4e^{-n\eta }, \end{array}$$

*where for each t=1,2,⋯,n, the probability distribution $Q_{X_{t}}$ on X appearing in (16) and the stochastic matrix ${\tilde{Q}}_{X_{t}|SX^{t-1}}:$$M_{1}\times X^{t-1}$→X appearing in (17) have a property that we can choose their values arbitrary.*

**Proof.**  In (12) in Lemma 1, we choose ${\hat{q}}_{SX^{n}Y^{n}}$ having the form

$$\begin{array}{c} {\hat{q}}_{SX^{n}Y^{n}}\left(S,X^{n},Y^{n}\right)=p_{S}\left(S\right)\prod_{t=1}^{n}\left\{p_{X_{t}|SX^{t-1}Y^{t}}\left(X_{t}|S,X^{t-1},Y^{t}\right)p_{Y_{t}|SY^{t-1}}\left(Y_{t}|S,Y^{t-1}\right)\right\}. \end{array}$$

In (13) in Lemma 1, we choose $Q_{X^{n}}$ having the form

$$Q_{X^{n}}\left(X^{n}\right)=\prod_{t=1}^{n}Q_{X_{t}}\left(X_{t}\right).$$

We further note that

$$\begin{array}{c} \frac{{\tilde{Q}}_{X^{n}|S}\left(X^{n}|S\right)}{p_{X^{n}}\left(X^{n}\right)}=\prod_{t=1}^{n}\frac{{\tilde{Q}}_{X_{t}|SX^{t-1}}\left(X_{t}|S,X^{t-1}\right)}{p_{X_{t}}\left(X_{t}\right)},p_{Y^{n}|S}\left(Y^{n}|S\right)=\prod_{t=1}^{n}p_{Y_{t}|SY^{t-1}}\left(Y_{t}|S,Y^{t-1}\right). \end{array}$$

Then, the bound (15) in Lemma 1 becomes

$$\begin{array}{cc} P_{c}^{\left(n\right)}\left(\varphi_{1}^{\left(n\right)},\varphi_{2}^{\left(n\right)},\psi^{\left(n\right)}\right)\leq p_{SX^{n}Y^{n}}\{ & \;\;\;0\geq \frac{1}{n}\sum_{t=1}^{n}\log\frac{p_{Y_{t}|SY^{t-1}}\left(Y_{t}|S,Y^{t-1}\right)}{p_{Y_{t}|SX^{t-1}Y^{t-1}}\left(Y_{t}|S,X^{t-1},Y^{t-1}\right)}-\eta ,\\ & \;\;\;0\geq \frac{1}{n}\sum_{t=1}^{n}\log\frac{Q_{X_{t}}\left(X_{t}\right)}{p_{X_{t}}\left(X_{t}\right)}-\eta ,\\ & R_{1}\geq \frac{1}{n}\sum_{t=1}^{n}\log\frac{{\tilde{Q}}_{X_{t}|SX^{t-1}}\left(X_{t}|S,X^{t-1}\right)}{p_{X_{t}}\left(X_{t}\right)}-\eta ,\\ & R_{2}\geq \left.\frac{1}{n}\sum_{t=1}^{n}\frac{1}{p_{Y_{t}|SY^{t-1}}\left(Y_{t}|S,Y^{t-1}\right)}-\eta \right\}+4e^{-n\eta }\\ & \leq p_{SX^{n}Y^{n}}\left\{0\geq \frac{1}{n}\sum_{t=1}^{n}\log\frac{Q_{X_{t}}\left(X_{t}\right)}{p_{X_{t}}\left(X_{t}\right)}-\eta ,\right.\\ & R_{1}\geq \frac{1}{n}\sum_{t=1}^{n}\log\frac{{\tilde{Q}}_{X_{t}|SX^{t-1}}\left(X_{t}|S,X^{t-1}\right)}{p_{X_{t}}\left(X_{t}\right)}-\eta ,\\ & R_{2}\geq \left.\frac{1}{n}\sum_{t=1}^{n}\log\frac{1}{p_{Y_{t}|SX^{t-1}Y^{t-1}}\left(Y_{t}|S,X^{t-1},Y^{t-1}\right)}-2\eta \right\}+4e^{-n\eta }, \end{array}$$

completing the proof. □

As in the standard converse coding argument, we identify auxiliary random variables, based on the bound in Lemma 2. The following lemma is necessary for such identification.

**Lemma** **3.** *Suppose that, for each t=1,2,⋯,n, the joint distribution $p_{SX^{t}Y^{t}}$ of the random vector $SX^{t}Y^{t}$ is a marginal distribution of $p_{SX^{n}Y^{n}}$. Then, we have the following Markov chain:*

(18) $$SX^{t-1}↔X_{t}↔Y_{t}$$

*or equivalently that $I(Y_{t};SX^{t-1}|X_{t})=0$. Furthermore, we have the following Markov chain:*

(19) $$Y^{t-1}↔SX^{t-1}↔\left(X_{t},Y_{t}\right)$$

*or equivalently that $I(X_{t}Y_{t};Y^{t-1}|SX^{t-1})=0$. The above two Markov chains are equivalent to the following one long Markov chain:*

(20) $$Y^{t-1}↔SX^{t-1}↔X_{t}↔Y_{t}.$$

Proof of this lemma is given in Appendix G. For t=1,2,⋯,n, set $U_{t}:=M_{1}$$\times X^{t-1}$. Define a random variable $U_{t}\in U_{t}$ by $U_{t}:=\left(S,X^{t-1}\right)$. From Lemmas 2 and 3, we identify auxiliary random variables to obtain the following lemma.

**Lemma** **4.** *For any η>0 and for any $(\varphi_{1}^{\left(n\right)}$, $\varphi_{2}^{\left(n\right)},\psi^{\left(n\right)})$ satisfying $\left(1/n\right)\log||\varphi_{i}^{\left(n\right)}\left|\right|\leq R_{i},i=1,2,$ we have*

(21) $$\begin{array}{c} P_{c}^{\left(n\right)}\left(\varphi_{1}^{\left(n\right)},\varphi_{2}^{\left(n\right)},\psi^{\left(n\right)}\right)\leq p_{SX^{n}Y^{n}}\left\{0\geq \frac{1}{n}\sum_{t=1}^{n}\log\frac{Q_{X_{t}}\left(X_{t}\right)}{p_{X_{t}}\left(X_{t}\right)}-\eta ,\right. \end{array}$$

(22) $$\begin{array}{c} R_{1}\geq \frac{1}{n}\sum_{t=1}^{n}\log\frac{{\tilde{Q}}_{X_{t}|U_{t}}\left(X_{t}|U_{t}\right)}{p_{X_{t}}\left(X_{t}\right)}-\eta , \end{array}$$

(23) $$\begin{array}{c} \left.R_{2}\geq \frac{1}{n}\sum_{t=1}^{n}\log\frac{1}{p_{Y_{t}|U_{t}}\left(Y_{t}|U_{t}\right)}-2\eta \right\}+4e^{-n\eta }, \end{array}$$

*where, for each t=1,2,⋯,n, the probability distribution $Q_{X_{t}}$ on X appearing in (21) and the stochastic matrix ${\tilde{Q}}_{X_{t}|U_{t}}:$$U_{t}$→X appearing in (22) have a property that we can choose their values arbitrary.*

Now, the challenge is that, although the quantities inside the first term in the right members of (23) in Lemma 4 have *n* sum of information spectrum quantities, the measure $p_{SX^{n}Y^{n}}$ does not have an i.i.d. structure in general. To resolve this, we first use the large deviation theory to upper bound the first quantity in the right members of (23). For each t=1,2,⋯,n, set ${\underline{Q}}_{t}:=\left(Q_{X_{t}},{\tilde{Q}}_{X_{t}|U_{t}}\right).$ Let ${\underline{Q}}_{t}$ be a set of all ${\underline{Q}}_{t}$. We define a quantity which serves as an exponential upper bound of $P_{c}^{\left(n\right)}(\varphi_{1}^{\left(n\right)},$ $\varphi_{2}^{\left(n\right)},\psi^{\left(n\right)})$. Let $P^{\left(n\right)}\left(p_{XY}\right)$ be a set of all probability distributions $p_{SX^{n}Y^{n}}$ on $M_{1}$ $\times X^{n}$ $\times Y^{n}$ having a form:

$$\begin{array}{c} p_{SX^{n}Y^{n}}\left(s,x^{n},y^{n}\right)=p_{S|X^{n}}\left(s|x^{n}\right)\prod_{t=1}^{n}p_{XY}\left(x_{t},y_{t}\right)\\ \;\mathrm{for}\;\left(s,x^{n},y^{n}\right)\in M_{1}\times X^{n}\times Y^{n}. \end{array}$$

For simplicity of notation, we use the notation $p^{\left(n\right)}$ for $p_{SX^{n}Y^{n}}$$\in P^{\left(n\right)}$$\left(p_{XY}\right)$. For each t=1,2,⋯,n, $p_{U_{t}X_{t}Y_{t}}=p_{SX^{t}Y_{t}}$ is a marginal distribution of $p^{\left(n\right)}$. For t=1,2,⋯,n, we simply write $p_{t}=$ $p_{U_{t}X_{t}Y_{t}}$. For μ∈[0,1], α∈[0,1), $p^{\left(n\right)}$ $\in P^{\left(n\right)}\left(p_{XY}\right)$, and ${\underline{Q}}^{n}$$\in Q^{n}$, we define

$$\begin{array}{c} \Omega^{\left(\mu ,\alpha \right)}\left(p^{\left(n\right)},{\underline{Q}}^{n}|p_{XY}\right):=-\log E_{p^{\left(n\right)}}\left[\prod_{t=1}^{n}\frac{p_{X_{t}}^{\bar{\alpha }}\left(X_{t}\right)}{Q_{X_{t}}^{\bar{\alpha }}\left(X_{t}\right)}\frac{p_{X_{t}}^{\mu \alpha }\left(X_{t}\right)p_{Y_{t}|U_{t}}^{\mu \alpha }\left(Y_{t}|U_{t}\right)}{{\tilde{Q}}_{X_{t}|U_{t}}^{\mu \alpha }\left(X_{t}|U_{t}\right)}\right], \end{array}$$

where for each t=1,2,⋯,n, the probability distribution $Q_{X_{t}}$ and the conditional probability distribution ${\tilde{Q}}_{X_{t}|U_{t}}$ appearing in the definition of $\Omega^{\left(\mu ,\theta \right)}\left(p^{\left(n\right)},{\underline{Q}}^{n}\right)$ can be chosen as arbitrary.

The following is well known as the Cramèr’s bound in the large deviation principle.

**Lemma** **5.** *For any real valued random variable Z and any α≥0, we have*

$$\mathrm{Pr}\left\{Z\geq a\right\}\leq \exp\left[-\left(\alpha a-\log E[\exp(\alpha Z)]\right)\right].$$

By Lemmas 4 and 5, we have the following proposition.

**Proposition** **1.** *For any $\left(\mu ,\alpha \right)\in {\left[0,1\right]}^{2}$ any ${\underline{Q}}^{n}\in {\underline{Q}}^{n}$, and any $(\varphi_{1}^{\left(n\right)},$$\varphi_{2}^{\left(n\right)},\psi^{\left(n\right)})$ satisfying $\left(1/n\right)\log\left|\right|\varphi_{i}^{\left(n\right)}\left|\right|\leq R_{i},i=1,2,$ there exists $p^{\left(n\right)}\in P^{\left(n\right)}\left(W_{1},W_{2}\right)$ such that*

$$\begin{array}{c} P_{c}^{\left(n\right)}\left(\varphi_{1}^{\left(n\right)},\varphi_{2}^{\left(n\right)},\psi^{\left(n\right)}\right)\leq 5\exp\left\{-n{\left[2+\alpha \bar{\mu }\right]}^{-1}\left[\frac{1}{n}\Omega^{\left(\mu ,\alpha \right)}\left(p^{\left(n\right)},{\underline{Q}}^{n}|p_{XY}\right)-\alpha \left(\mu R_{1}+\bar{\mu }R_{2}\right)\right]\right\}. \end{array}$$

**Proof.**  By Lemma 4, for $\left(\mu ,\alpha \right)\in {\left[0,1\right]}^{2}$, we have the following chain of inequalities:

(24) $$\begin{array}{c} P_{c}^{\left(n\right)}\left(\varphi_{1}^{\left(n\right)},\varphi_{2}^{\left(n\right)},\psi^{\left(n\right)}\right)\leq p_{SX^{n}Y^{n}}\left\{0\geq \left[\frac{1}{n}\sum_{t=1}^{n}\log\frac{Q_{X_{t}}^{\bar{\alpha }}\left(X_{t}\right)}{p_{X_{t}}^{\bar{\alpha }}\left(X_{t}\right)}-\bar{\alpha }\eta \right],\right.\\ \;\alpha \mu R_{1}\geq \frac{1}{n}\sum_{t=1}^{n}\log\frac{{\tilde{Q}}_{X_{t}|U_{t}}^{\alpha \mu }\left(X_{t}|U_{t}\right)}{p_{X_{t}}^{\alpha \mu }\left(X_{t}\right)}-\alpha \mu \eta ,\\ \;\left.\alpha \bar{\mu }R_{2}\geq \frac{1}{n}\sum_{t=1}^{n}\log\frac{1}{p_{Y_{t}|U_{t}}^{\alpha \bar{\mu }}\left(Y_{t}|U_{t}\right)}-2\alpha \bar{\mu }\eta \right\}+4e^{-n\eta }\\ \leq p_{SX^{n}Y^{n}}\left\{\begin{array}{c} \;\\ \; \end{array}\right.\;\;\alpha \left(\mu R_{1}+\bar{\mu }R_{2}\right)+\left(1+\alpha \bar{\mu }\right)\eta \left.\geq -\frac{1}{n}\sum_{t=1}^{n}\log\left[\frac{p_{X_{t}}^{\bar{\alpha }}\left(X_{t}\right)}{Q_{X_{t}}^{\bar{\alpha }}\left(X_{t}\right)}\frac{p_{X_{t}}^{\mu \alpha }\left(X_{t}\right)p_{Y_{t}|U_{t}}^{\bar{\mu }\alpha }\left(Y_{t}|U_{t}\right)}{{\tilde{Q}}_{X_{t}|U_{t}}^{\mu \alpha }\left(X_{t}|U_{t}\right)}\right]\right\}+4e^{-n\eta }\\ =p_{SX^{n}Y^{n}}\left\{\frac{1}{n}\sum_{t=1}^{n}\log\left[\frac{p_{X_{t}}^{\bar{\alpha }}\left(X_{t}\right)}{Q_{X_{t}}^{\bar{\alpha }}\left(X_{t}\right)}\frac{p_{X_{t}}^{\mu \alpha }\left(X_{t}\right)p_{Y_{t}|U_{t}}^{\alpha }\left(Y_{t}|U_{t}\right)}{{\tilde{Q}}_{X_{t}|U_{t}}^{\mu \alpha }\left(X_{t}|U_{t}\right)}\right]\geq -\left[\alpha \left(\mu R_{1}+\bar{\mu }R_{2}\right)+\left(1+\alpha \bar{\mu }\right)\eta \right]\right\}+4e^{-n\eta }\\ \overset{\left(a\right)}{\leq }\exp\left[n\left\{\alpha \left(\mu R_{1}+\bar{\mu }R_{2}\right)+\left(1+\alpha \bar{\mu }\right)\eta -\frac{1}{n}\Omega^{\left(\mu ,\alpha \right)}\left(p^{\left(n\right)},{\underline{Q}}^{n}|p_{XY}\right)\right\}\right]+4e^{-n\eta }. \end{array}$$

Step (a) follows from Lemma 5. When $\Omega^{\left(\mu ,\alpha \right)}\left(p^{\left(n\right)},{\underline{Q}}^{n}|p_{XY}\right)\leq n\alpha \left(\mu R_{1}+\bar{\mu }R_{2}\right)$, the bound we wish to prove is obvious. In the following argument, we assume that $\Omega^{\left(\mu ,\alpha \right)}\left(p^{\left(n\right)},{\underline{Q}}^{n}|p_{XY}\right)>$ $n\alpha (\mu R_{1}+\bar{\mu }R_{2})$. We choose η so that

(25) $$\begin{array}{ccc} -\eta & = & \alpha \left(\mu R_{1}+\bar{\mu }R_{2}\right)+\left(1+\alpha \bar{\mu }\right)\eta -\frac{1}{n}\Omega^{\left(\mu ,\alpha \right)}\left(p^{\left(n\right)},{\underline{Q}}^{n}|p_{XY}\right). \end{array}$$

Solving (25) with respect to η, we have

$$\begin{array}{c} \eta =\frac{\left(1/n\right)\Omega^{\left(\mu ,\alpha \right)}\left(p^{\left(n\right)},{\underline{Q}}^{n}|p_{XY}\right)-\alpha \left(\mu R_{1}+\bar{\mu }R_{2}\right)}{2+\alpha \bar{\mu }}. \end{array}$$

For this choice of η and (24), we have

$$\begin{array}{c} P_{c}^{\left(n\right)}\left(\varphi_{1}^{\left(n\right)},\varphi_{2}^{\left(n\right)},\psi^{\left(n\right)}\right)\leq 5e^{-n\eta }=5\exp\left\{-n{\left[2+\alpha \bar{\mu }\right]}^{-1}\left[\frac{1}{n}\Omega^{\left(\mu ,\alpha \right)}\left(p^{\left(n\right)},{\underline{Q}}^{n}|p_{XY}\right)-\alpha \left(\mu R_{1}+\bar{\mu }R_{2}\right)\right]\right\}, \end{array}$$

completing the proof. □

Set

$$\begin{array}{c} {\underline{\Omega }}^{\left(\mu ,\alpha \right)}\left(p_{XY}\right):=\underset{n\geq 1}{\inf}\min_{p^{\left(n\right)}\in P^{\left(n\right)}}\max_{{\underline{Q}}^{n}\in {\underline{Q}}^{n}}\frac{1}{n}\Omega^{\left(\mu ,\alpha \right)}\left(p^{\left(n\right)},{\underline{Q}}^{n}|p_{XY}\right). \end{array}$$

By Proposition 1, we have the following corollary.

**Corollary** **3.** *For any $\left(\mu ,\alpha \right)\in {\left[0,1\right]}^{2}$ and any $(\varphi_{1}^{\left(n\right)},$$\varphi_{2}^{\left(n\right)},\psi^{\left(n\right)})$ satisfying $\left(1/n\right)\log\left|\right|\varphi_{i}^{\left(n\right)}\left|\right|\leq R_{i},i=1,2,$ we have*

$$\begin{array}{c} P_{c}^{\left(n\right)}\left(\varphi_{1}^{\left(n\right)},\varphi_{2}^{\left(n\right)},\psi^{\left(n\right)}\right)\leq 5\exp\left\{-n\left[\frac{{\underline{\Omega }}^{\left(\mu ,\alpha \right)}\left(p_{XY}\right)-\alpha \left(\mu R_{1}+\bar{\mu }R_{2}\right)}{2+\alpha \bar{\mu }}\right]\right\}. \end{array}$$

We shall call ${\underline{\Omega }}^{\left(\mu ,\alpha \right)}\left(p_{XY}\right)$ the communication potential. The above corollary implies that the analysis of ${\underline{\Omega }}^{\left(\mu ,\alpha \right)}($$p_{XY})$ leads to an establishment of a strong converse theorem for the one helper source coding problem. Note here that ${\underline{\Omega }}^{\left(\mu ,\alpha \right)}\left(p_{XY}\right)$ is still a multi letter quantity. However, we successfully single letterize this quantity. This result which will be stated later in Proposition 2 is a mathematical core of our main result.

In the following argument, we drive an explicit lower bound of ${\underline{\Omega }}^{\left(\mu ,\alpha \right)}\left(p_{XY}\right)$. For each t=1,2,⋯,n, set $u_{t}=\left(s,x^{t-1}\right)$$\in U_{t}$ and

$$F_{t}:=\left(p_{X_{t}},p_{X_{t}Y_{t}|U_{t}},{\underline{Q}}_{t}\right),\;F^{t}:={\left\{F_{i}\right\}}_{i=1}^{t}.$$

For t=1,2,⋯,n, define a function of $\left(u_{t},x_{t},y_{t}\right)$ $\in U_{t}$ ×X ×Y by

$$\begin{array}{c} f_{F_{t}}^{\left(\mu ,\alpha \right)}\left(x_{t},y_{t}|u_{t}\right):=\frac{p_{X_{t}}^{\bar{\alpha }}\left(x_{t}\right)}{Q_{X_{t}}^{\bar{\alpha }}\left(x_{t}\right)}\frac{p_{X_{t}}^{\mu \alpha }\left(x_{t}\right)p_{Y_{t}|U_{t}}^{\alpha }\left(y_{t}|u_{t}\right)}{{\tilde{Q}}_{X_{t}|U_{t}}^{\mu \alpha }\left(x_{t}|u_{t}\right)}. \end{array}$$

By definition, we have

$$\begin{array}{c} \exp\left\{-\Omega^{\left(\mu ,\alpha \right)}\left(p^{\left(n\right)},{\underline{Q}}^{n}|p_{XY}\right)\right\}=\sum_{s,x^{n},y^{n}}p_{SX^{n}Y^{n}}\left(s,x^{n},y^{n}\right)\prod_{t=1}^{n}f_{F_{t}}^{\left(\mu ,\alpha \right)}\left(x_{t},y_{t}|u_{t}\right). \end{array}$$

For each t=1,2,⋯,n, we define the probability distribution

$$p_{SX^{t}Y^{t};F^{t}}^{\left(\mu ,\alpha \right)}:={\left\{p_{SX^{t}Y^{t};F^{t}}^{\left(\mu ,\alpha \right)}\left(s,x^{t},y^{t}\right)\right\}}_{\left(s,x^{t},y^{t}\right)\in M_{1}\times X^{t}\times Y^{t}}$$

by

$$\begin{array}{c} p_{SX^{t}Y^{t};F^{t}}^{\left(\mu ,\alpha \right)}\left(s,x^{t},y^{t}\right):=C_{t}^{-1}p_{SX^{t}Y^{t}}\left(s,x^{t},y^{t}\right)\prod_{i=1}^{t}f_{F_{i}}^{\left(\mu ,\alpha \right)}\left(x_{i},y_{i}|u_{i}\right), \end{array}$$

where

$$\begin{array}{cc} C_{t} & :=\sum_{s,x^{t},y^{t}}p_{SX^{t}Y^{t}}\left(s,x^{t},y^{t}\right)\prod_{i=1}^{t}f_{F_{i}}^{\left(\mu ,\alpha \right)}\left(x_{i},y_{i}\right) \end{array}$$

are constants for normalization. For t=1,2,⋯,n, define

(26) $$\Phi_{t}^{\left(\mu ,\alpha \right)}:=C_{t}C_{t-1}^{-1},$$

where we define $C_{0}=1$. Then, we have the following lemma.

**Lemma** **6.** *For each t=1,2,⋯,n, and for any (s,$x^{t},y^{t})\in M_{1}$$\times X^{t}$$\times Y^{t}$, we have*

(27) $$\begin{array}{c} p_{SX^{t}Y^{t};F^{t}}^{\left(\mu ,\alpha \right)}\left(s,x^{t},y^{t}\right)\\ ={\left(\Phi_{t}^{\left(\mu ,\alpha \right)}\right)}^{-1}p_{SX^{t-1}Y^{t-1};F^{t-1}}^{\left(\mu ,\alpha \right)}\left(s,x^{t-1},y^{t-1}\right)p_{X_{t}Y_{t}|SX^{t-1}Y^{t-1}}\left(x_{t},y_{t}|s,x^{t-1},y^{t-1}\right)f_{F_{t}}^{\left(\mu ,\alpha \right)}\left(x_{t},y_{t}|u_{t}\right). \end{array}$$

*Furthermore, we have*

(28) $$\begin{array}{c} \Phi_{t}^{\left(\mu ,\alpha \right)}=\sum_{s,x^{t},y^{t}}p_{SX^{t-1}Y^{t-1};F^{t-1}}^{\left(\mu ,\alpha \right)}\left(s,x^{t-1},y^{t-1}\right)p_{X_{t}Y_{t}|SX^{t-1}Y^{t-1}}\left(x_{t},y_{t}|s,x^{t-1},y^{t-1}\right)f_{F_{t}}^{\left(\mu ,\alpha \right)}\left(x_{t},y_{t}|u_{t}\right). \end{array}$$

Proof of this lemma is given in Appendix H. Define

$$\begin{array}{c} p_{U_{t};F^{t-1}}^{\left(\mu ,\alpha \right)}\left(u_{t}\right)=p_{SX^{t-1};F^{t-1}}^{\left(\mu ,\alpha \right)}\left(s,x^{t-1}\right):=\sum_{y^{t-1}}p_{SX^{t-1}Y^{t-1};F^{t-1}}^{\left(\mu ,\alpha \right)}\left(s,x^{t-1},y^{t-1}\right). \end{array}$$

Then, we have the following lemma, which is a key result to derive a single letterized lower bound of ${\underline{\Omega }}^{\left(\mu ,\alpha \right)}\left(p_{XY}\right)$.

**Lemma** **7.** *For any $p^{\left(n\right)}\in P^{\left(n\right)}\left(p_{XY}\right)$ and any ${\underline{Q}}^{n}\in {\underline{Q}}^{n}$, we have*

(29) $$\begin{array}{c} \Omega^{\left(\mu ,\alpha \right)}\left(p^{\left(n\right)},{\underline{Q}}^{n}|p_{XY}\right)=\left(-1\right)\sum_{t=1}^{n}\log\Phi_{t}^{\left(\mu ,\alpha \right)}, \end{array}$$

(30) $$\begin{array}{c} \Phi_{t}^{\left(\mu ,\alpha \right)}=\sum_{u_{t},x_{t},y_{t}}p_{U_{t};F^{t-1}}^{\left(\mu ,\alpha \right)}\left(u_{t}\right)p_{X_{t}|U_{t}}\left(x_{t}|u_{t}\right)p_{Y_{t}|X_{t}}\left(y_{t}|x_{t}\right)f_{F_{t}}^{\left(\mu ,\alpha \right)}\left(x_{t},y_{t}|u_{t}\right). \end{array}$$

**Proof.**  We first prove (29). From (26), we have

(31) $$\log\Phi_{t}^{\left(\mu ,\alpha \right)}=-\log C_{t}+\log C_{t-1}.$$

Furthermore, by definition, we have

(32) $$\Omega^{\left(\mu ,\alpha \right)}\left(p^{\left(n\right)},{\underline{Q}}^{n}|p_{XY}\right)=-\log C_{n},C_{0}=1.$$

From (31) and (32), (29) is obvious. We next prove (30). We first observe that for $\left(s,x^{t},y^{t}\right)$ $\in S\times X^{t}\times Y^{t}$ and for t=1,2,⋯,n,

$$\begin{array}{c} p_{X_{t}Y_{t}|SX^{t-1}Y^{t-1}}\left(x_{t},y_{t}|s,x^{t-1},y^{t-1}\right)=p_{X_{t}|SX^{t-1}Y^{t-1}}\left(x_{t}|s,x^{t-1},y^{t-1}\right)p_{Y_{t}|SX^{t}Y^{t-1}}\left(y_{t}|s,x^{t},y^{t-1}\right)\\ \overset{\left(a\right)}{=}p_{X_{t}|SX^{t-1}}\left(x_{t}|s,x^{t-1}\right)p_{Y_{t}|X_{t}}\left(y_{t}|x_{t}\right). \end{array}$$

Step (a) follows from Lemma 3. Then, by Lemma 6, we have

$$\begin{array}{c} \Phi_{t}^{\left(\mu ,\alpha \right)}=\sum_{s,x^{t},y^{t}}p_{SX^{t-1}Y^{t-1};F^{t-1}}^{\left(\mu ,\alpha \right)}\left(s,x^{t-1},y^{t-1}\right)p_{X_{t}Y_{t}|SX^{t-1}Y^{t-1}}\left(x_{t},y_{t}|s,x^{t-1},y^{t-1}\right)f_{F_{t}}^{\left(\mu ,\alpha \right)}\left(x_{t},y_{t}|u_{t}\right)\\ =\sum_{s,x^{t},y_{t}}\left\{\sum_{y^{t-1}}p_{SX^{t-1}Y^{t-1};F^{t-1}}^{\left(\mu ,\alpha \right)}\left(s,x^{t-1},y^{t-1}\right)\right\}p_{X_{t}|SX^{t-1}}\left(x_{t}|s,x^{t-1}\right)p_{Y_{t}|X_{t}}\left(y_{t}|x_{t}\right)f_{F_{t}}^{\left(\mu ,\alpha \right)}\left(x_{t},y_{t}|u_{t}\right)\\ =\sum_{s,x^{t},y_{t}}p_{SX^{t-1};F^{t-1}}^{\left(\mu ,\alpha \right)}\left(s,x^{t-1}\right)p_{X_{t}|SX^{t-1}}\left(x_{t}|s,x^{t-1}\right)p_{Y_{t}|X_{t}}\left(y_{t}|x_{t}\right)f_{F_{t}}^{\left(\mu ,\alpha \right)}\left(x_{t},y_{t}|u_{t}\right), \end{array}$$

completing the proof. □

The following proposition is a mathematical core to prove our main result.

**Proposition** **2.** *For any μ∈[0,1] and any α≥0, we have*

$${\underline{\Omega }}^{\left(\mu ,\alpha \right)}\left(p_{XY}\right)\geq \Omega^{\left(\mu ,\alpha \right)}\left(p_{XY}\right).$$

**Proof.**  Set

Qn(pY|X):={q=qUXY:|U|≤|M1||Xn−1||Yn−1|,qY|X=pY|X,U↔X↔Y},Ω^n(μ,α)(pXY):=minq∈Qn(pY|X)Ω(μ,α)(q|pXY).

For each t=1,2,⋯,n, we define $q_{t}=q_{U_{t}X_{t}Y_{t}Z_{t}}$ by

(33) $$\begin{array}{c} q_{U_{t}}\left(u_{t}\right)=p_{U_{t};F^{t-1}}^{\left(\mu ,\alpha \right)}\left(u_{t}\right),q_{X_{t}Y_{t}|U_{t}}\left(x_{t},y_{t}|u_{t}\right)=p_{X_{t}|U_{t}}\left(x_{t}|u_{t}\right)p_{Y|X}\left(y_{t}|x_{t}\right). \end{array}$$

Equation (33) implies that $q_{t}=q_{U_{t}X_{t}Y_{t}}\in Q_{n}\left(p_{Y|X}\right).$ Furthermore, for each t=1,2,⋯,n, we choose ${\underline{Q}}_{t}=\left(Q_{X_{t}},{\tilde{Q}}_{X_{t}|U_{t}}\right)$ appearing in

$$\begin{array}{c} f_{F_{t}}^{\left(\mu ,\alpha \right)}\left(x_{t},y_{t}|u_{t}\right)=\frac{p_{X_{t}}^{\bar{\alpha }}\left(x_{t}\right)}{Q_{X_{t}}^{\bar{\alpha }}\left(x_{t}\right)}\frac{p_{X_{t}}^{\mu \alpha }\left(x_{t}\right)p_{Y_{t}|U_{t}}^{\alpha }\left(y_{t}|u_{t}\right)}{{\tilde{Q}}_{X_{t}|U_{t}}^{\mu \alpha }\left(x_{t}|u_{t}\right)} \end{array}$$

such that ${\underline{Q}}_{t}=\left(Q_{X_{t}},{\tilde{Q}}_{X_{t}|U_{t}}\right)=\left(q_{X_{t}},q_{X_{t}|U_{t}}\right).$ For this choice of ${\underline{Q}}_{t}$, we have the following chain of inequalities:

(34) $$\begin{array}{c} \Phi_{t}^{\left(\mu ,\alpha \right)}\overset{\left(a\right)}{=}E_{q_{t}}\left[f_{F_{t}}^{\left(\mu ,\theta \right)}\left(X_{t},Y_{t}|U_{t}\right)\right]\overset{\left(b\right)}{=}E_{q_{t}}\left[\frac{p_{X_{t}}^{\bar{\alpha }}\left(X_{t}\right)}{q_{X_{t}}^{\bar{\alpha }}\left(X_{t}\right)}\frac{p_{X_{t}}^{\mu \alpha }\left(X_{t}\right)p_{Y_{t}|U_{t}}^{\alpha }\left(Y_{t}|U_{t}\right)}{q_{X_{t}|U_{t}}^{\mu \alpha }\left(X_{t}|U_{t}\right)}\right]=E_{q_{t}}\left[f_{q_{t}|p_{X_{t}}}^{\left(\mu ,\alpha \right)}\left(X_{t},Y_{t}|U_{t}\right)\right]\\ =\exp\left\{-\Omega^{\left(\mu ,\alpha \right)}\left(q_{t}|p_{X_{t}}\right)\right\}\overset{\left(c\right)}{=}\exp\left\{-\Omega^{\left(\mu ,\alpha \right)}\left(q_{t}|p_{X}\right)\right\}\\ \overset{\left(d\right)}{\leq }\exp\left\{-{\hat{\Omega }}_{n}^{\left(\mu ,\alpha \right)}\left(p_{XY}\right)\right\}\overset{\left(e\right)}{=}\exp\left\{-\Omega^{\left(\mu ,\alpha \right)}\left(p_{XY}\right)\right\}. \end{array}$$

Step (a) follows from Lemma 7 and (33). Step (b) follows from the choice $(Q_{X_{t}},{\tilde{Q}}_{X_{t}|U_{t}})=$ $\left(q_{X_{t}},q_{X_{t}|U_{t}}\right)$ of $\left(Q_{X_{t}},{\tilde{Q}}_{X_{t}|U_{t}}\right)$ for t=1,2,⋯,n. Step (c) follows from $p_{X_{t}}=p_{X}$ for t=1,2,⋯,n. Step (d) follows from $q_{t}\in Q_{n}\left(p_{Y|X}\right)$ and the definition of ${\hat{\Omega }}_{n}^{\left(\mu ,\alpha \right)}\left(p_{XY}\right)$. Step (e) follows from Property 4 part a. Hence, we have the following:

(35) $$\begin{array}{c} \max_{{\underline{Q}}^{n}\in {\underline{Q}}^{n}}\frac{1}{n}\Omega^{\left(\mu ,\alpha \right)}\left(p^{\left(n\right)},{\underline{Q}}^{n}|p_{XY}\right)\geq \frac{1}{n}\Omega^{\left(\mu ,\alpha \right)}\left(p^{\left(n\right)},{\underline{Q}}^{n}|p_{XY}\right)\overset{\left(a\right)}{=}-\frac{1}{n}\sum_{t=1}^{n}\log\Phi_{t}^{\left(\mu ,\alpha \right)}\overset{\left(b\right)}{\geq }\Omega^{\left(\mu ,\alpha \right)}\left(p_{XY}\right). \end{array}$$

Step (a) follows from Lemma 7. Step (b) follows from (34). Since (35) holds fo any n≥1 and any $p_{SX^{n}Y^{n}}$ satisfying $S↔X^{n}↔Y^{n}$, we have that, for any $\left(\mu ,\alpha \right)\in {\left[0,1\right]}^{2}$,

$${\underline{\Omega }}^{\left(\mu ,\alpha \right)}\left(p_{XY}\right)\geq \Omega^{\left(\mu ,\alpha \right)}\left(p_{XY}\right).$$

Thus, Proposition 2 is proved. □

**Proof** **of** **Theorem** **3.** For any $\left(\mu ,\alpha \right)\in {\left[0,1\right]}^{2}$, for any $R_{1},R_{2}\geq 0$ and for any $(\varphi_{1}^{\left(n\right)},$$\varphi_{2}^{\left(n\right)},$ $\psi^{\left(n\right)})$ satisfying $\left(1/n\right)\log||\varphi_{i}^{\left(n\right)}\left|\right|\leq R_{i},i=1,2,$ we have the following:

$$\begin{array}{c} \frac{1}{n}\log\left\{\frac{5}{P_{c}^{\left(n\right)}\left(\varphi_{1}^{\left(n\right)},\varphi_{2}^{\left(n\right)},\psi^{\left(n\right)}\right)}\right\}\overset{\left(a\right)}{\geq }\frac{{\underline{\Omega }}^{\left(\mu ,\alpha \right)}\left(p_{XY}\right)-\alpha \left(\mu R_{1}+\bar{\mu }R_{2}\right)}{2+\alpha \bar{\mu }}\overset{\left(b\right)}{\geq }\frac{\Omega^{\left(\mu ,\alpha \right)}\left(p_{XY}\right)-\alpha \left(\mu R_{1}+\bar{\mu }R_{2}\right)}{2+\alpha \bar{\mu }}\\ =F^{\left(\mu ,\alpha \right)}\left(\mu R_{1}+\bar{\mu }R_{2}|p_{XY}\right). \end{array}$$

Step (a) follows from Corollary 3. Step (b) follows from Proposition 2. Since the above bound holds for any μ∈[0,1] and any α≥0, we have

$$\frac{1}{n}\log\left\{\frac{5}{P_{c}^{\left(n\right)}\left(\varphi_{1}^{\left(n\right)},\varphi_{2}^{\left(n\right)},\psi^{\left(n\right)}\right)}\right\}\geq F\left(R_{1},R_{2}|p_{XY}\right).$$

Thus, (10) in Theorem 3 is proved. □

**Proof.** **of Corollary 2.** Since *g* is an inverse function of ϑ, the definition (11) of $\kappa_{n}$ is equivalent to

(36) $$g\left(\frac{\kappa_{n}}{\rho (p_{XY})}\right)=\sqrt{\frac{4}{n\rho (p_{XY})}\log\left(\frac{5}{1-\varepsilon }\right)}.$$

By the definition of $n_{0}=n_{0}\left(\varepsilon ,\rho \left(p_{XY}\right)\right)$, we have that $\kappa_{n}\leq \left(1/2\right)\rho \left(p_{XY}\right)$ for $n\geq n_{0}$. We assume that, for $n\geq n_{0}$, $\left(R_{1},R_{2}\right)\in R_{\mathrm{AKW}}\left(n,\varepsilon |p_{XY}\right).$ Then, there exists a sequence $\{(\varphi_{1}^{\left(n\right)},$ $\varphi_{2}^{\left(n\right)},$ $\psi^{\left(n\right)})$ ${\}}_{n\geq n_{0}}$ such that, for $n\geq n_{0}$, we have

(37) $$\begin{array}{c} \frac{1}{n}\log||\varphi_{i}^{\left(n\right)}\left|\right|\leq R_{i},i=1,2,1-\varepsilon \leq P_{c}^{\left(n\right)}\left(\varphi_{1}^{\left(n\right)},\varphi_{2}^{\left(n\right)},\psi^{\left(n\right)}\right). \end{array}$$

Then, by Theorem 3, we have

(38) $$\begin{array}{ccc} 1-\varepsilon & \leq & P_{c}^{\left(n\right)}\left(\varphi_{1}^{\left(n\right)},\varphi_{2}^{\left(n\right)},\psi^{\left(n\right)}\right)\leq 5\exp\left\{-nF(R_{1},R_{2}|p_{XY})\right\} \end{array}$$

for any $n\geq n_{0}\left(\varepsilon ,\rho \left(p_{XY}\right)\right)$. From (38), we have that for $n\geq n_{0}(\varepsilon ,\rho ($$p_{XY}))$,

(39) $$\begin{array}{c} F\left(R_{1},R_{2}|p_{XY}\right)\leq \frac{1}{n}\log\left(\frac{5}{1-\varepsilon }\right)\overset{\left(a\right)}{=}\frac{\rho (p_{XY})}{4}\cdot g^{2}\left(\frac{\kappa_{n}}{\rho (p_{XY})}\right). \end{array}$$

Step (a) follows from (36). Hence, by Property 4 part e, we have that, under $\kappa_{n}\leq \left(1/2\right)\rho \left(p_{XY}\right)$, the inequality (39) implies

(40) $$\left(R_{1},R_{2}\right)\in R\left(p_{XY}\right)+\kappa_{n}\left(1,1\right).$$

Since (40) holds for any $n\geq n_{0}$ and $\left(R_{1},R_{2}\right)\in R_{\mathrm{AKW}}($$n,\varepsilon |p_{XY})$, we have

$$R_{\mathrm{AKW}}\left(n,\varepsilon |p_{XY}\right)\subseteq R\left(p_{XY}\right)+\kappa_{n}\left(1,1\right)\;\mathrm{for}\;n\geq n_{0},$$

completing the proof. □

## 5. One Helper Problem Studied by Wyner

**Figure 4:**
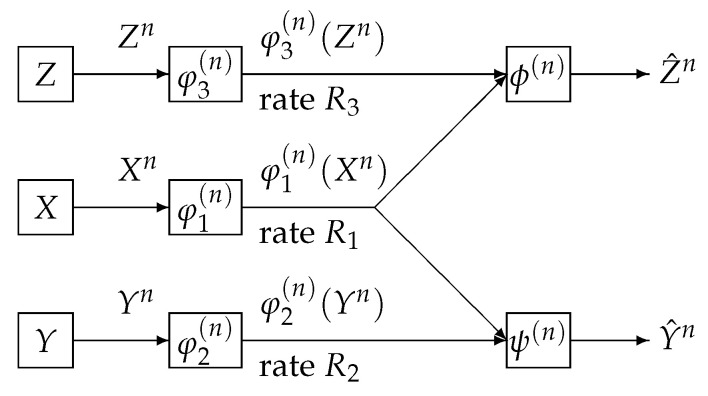
One helper source coding system investigated by Wyner.

We consider a communication system depicted in Figure 4. Data sequences $X^{n}$, $Y^{n}$, and $Z^{n}$, respectively are separately encoded to $\varphi_{1}^{\left(n\right)}\left(X^{n}\right)$, $\varphi_{2}^{\left(n\right)}\left(Y^{n}\right)$, and $\varphi_{3}^{\left(n\right)}\left(Z^{n}\right)$. The encoded data $\varphi_{1}^{\left(n\right)}\left(X^{n}\right)$ and $\varphi_{2}^{\left(n\right)}\left(Y^{n}\right)$ are sent to the information processing center 1. The encoded data $\varphi_{1}^{\left(n\right)}\left(X^{n}\right)$ and $\varphi_{3}^{\left(n\right)}\left(Z^{n}\right)$ are sent to the information processing center 2. At center 1, the decoder function $\psi^{\left(n\right)}$ observes $(\varphi_{1}^{\left(n\right)}\left(X^{n}\right),$$\varphi_{2}^{\left(n\right)}\left(Y^{n}\right))$ to output the estimation ${\hat{Y}}^{n}$ of $Y^{n}$. At center 2, the decoder function $\phi^{\left(n\right)}$ observes $(\varphi_{1}^{\left(n\right)}\left(X^{n}\right),$$\varphi_{3}^{\left(n\right)}\left(Z^{n}\right))$ to output the estimation ${\hat{Z}}^{n}$ of $Z^{n}$. The error probability of decoding is

$$\begin{array}{c} P_{e}^{\left(n\right)}\left(\varphi_{1}^{\left(n\right)},\varphi_{2}^{\left(n\right)},\varphi_{3}^{\left(n\right)},\psi^{\left(n\right)},\phi^{\left(n\right)}\right)=\mathrm{Pr}\left\{{\hat{Y}}^{n}\neq Y^{n}or{\hat{Z}}^{n}\neq Z^{n}\right\}, \end{array}$$

where ${\hat{Y}}^{n}=\psi^{\left(n\right)}($$\varphi_{1}^{\left(n\right)}\left(X^{n}\right),\varphi_{2}^{\left(n\right)}\left(Y^{n}\right))$ and ${\hat{Z}}^{n}=\psi^{\left(n\right)}($$\varphi_{1}^{\left(n\right)}\left(X^{n}\right),\varphi_{3}^{\left(n\right)}\left(Z^{n}\right))$.

A rate triple $\left(R_{1},R_{2},R_{3}\right)$ is ε-*achievable* if, for any δ>0, there exist a positive integer $n_{0}=n_{0}\left(\varepsilon ,\delta \right)$ and a sequence of three encoders and two decoder functions $\{(\varphi_{1}^{\left(n\right)},\varphi_{2}^{\left(n\right)},$$\varphi_{3}^{\left(n\right)},\psi^{\left(n\right)},$ $\phi^{\left(n\right)}{)\}}_{n\geq n_{0}}$ such that, for $n\geq n_{0}\left(\varepsilon ,\delta \right)$,

$$\begin{array}{c} \frac{1}{n}\log||\varphi_{i}^{\left(n\right)}\left|\right|\leq R_{i}+\delta fori=1,2,3,P_{e}^{\left(n\right)}\left(\varphi_{1}^{\left(n\right)},\varphi_{2}^{\left(n\right)},\varphi_{3}^{\left(n\right)},\psi^{\left(n\right)},\phi^{\left(n\right)}\right)\leq \varepsilon . \end{array}$$

The rate region $R_{W}\left(\varepsilon |p_{XYZ}\right)$ is defined by

$$\begin{array}{c} R_{W}\left(\varepsilon |p_{XYZ}\right):=\left\{\left(R_{1},R_{2},R_{3}\right):\left(R_{1},R_{2},R_{3}\right)is\varepsilon -\mathrm{achievable}\;\mathrm{for}p_{XYZ}\right\}. \end{array}$$

Furthermore, define

$$R_{W}\left(p_{XYZ}\right):=\underset{\varepsilon \in (0,1)}{⋂}R_{W}\left(\varepsilon |p_{XYZ}\right).$$

We can show that the two rate regions $R_{W}\left(\varepsilon \right|$$p_{XYZ})$, ε∈(0,1) and $R_{W}\left(p_{XYZ}\right)$ satisfy the following property.

**Property** **5.**

*(a)*  *The regions $R_{W}\left(\varepsilon |p_{XYZ}\right)$, ε∈(0,1), and $R_{W}($$p_{XYZ})$ are closed convex sets of $R_{+}^{3}$.*
*(b)*  *We set*
  $$\begin{array}{c} R_{W}\left(n,\varepsilon |p_{XYZ}\right)=\{\left(R_{1},R_{2},R_{3}\right):There\;exists\left(\varphi_{1}^{\left(n\right)},\varphi_{2}^{\left(n\right)},\varphi_{3}^{\left(n\right)},\psi^{\left(n\right)}\right)such\;that\\ \frac{1}{n}\log||\varphi_{i}^{\left(n\right)}\left|\right|\leq R_{i},i=1,2,3,P_{e}^{\left(n\right)}\left(\varphi_{1}^{\left(n\right)},\varphi_{2}^{\left(n\right)},\varphi_{3}^{\left(n\right)},\psi^{\left(n\right)}\right)\leq \varepsilon \}, \end{array}$$
  *which is called the (n,ε)-rate region. Using $R_{W}(n,$$\varepsilon |p_{XYZ})$, $R_{W}\left(\varepsilon |p_{XYZ}\right)$ can be expressed as*
  $$\begin{array}{cc} R_{W}\left(\varepsilon |p_{XYZ}\right) & =\mathrm{cl}\left(\underset{m\geq 1}{⋃}\underset{n\geq m}{⋂}R_{W}\left(n,\varepsilon |p_{XYZ}\right)\right). \end{array}$$

It is well known that $R_{W}\left(p_{XYZ}\right)$ was determined by Wyner. To describe his result, we introduce an auxiliary random variable *U* taking values in a finite set U. We assume that the joint distribution of (U,X,Y,Z) is

$$p_{UXY}\left(u,x,y,z\right)=p_{U}\left(u\right)p_{X|U}\left(x|u\right)p_{YZ|X}\left(y,z|x\right).$$

The above condition is equivalent to U↔X↔YZ. Define the set of probability distribution on U ×X ×Y ×Z by

$$\begin{array}{cc} P(p_{XYZ}):= & \{p=p_{UXYZ}:|U|\leq |X|+2,U↔X↔YZ\}. \end{array}$$

Set

$$\begin{array}{cc} R(p) & :=\begin{array}{c} \{\left(R_{1},R_{2},R_{3}\right):R_{1},R_{2},R_{3}\geq 0,\;\\ \begin{array}{ccc} R_{1} & \geq & I_{p}\left(X;U\right),R_{2}\geq H_{p}\left(Y|U\right),R_{3}\geq H_{p}\left(Z|U\right)\}, \end{array} \end{array}\\ R(p_{XYZ}) & :=\underset{p\in P(p_{XYZ})}{⋃}R\left(p\right). \end{array}$$

We can show that the region $R(p_{XYZ})$ satisfies the following property.

**Property** **6.**

*(a)*  *The region $R(p_{XYZ})$ is a closed convex subset of $R_{+}^{3}$.*
*(b)*  *For any $p_{XYZ}$, and any γ∈[0,1], we have*
  (41) $$\begin{array}{c} \min_{\left(R_{1},R_{2},R_{3}\right)\in R\left(p_{XY}\right)}\left(R_{1}+\bar{\gamma }R_{2}+\gamma R_{3}\right)=\bar{\gamma }H_{p}\left(Y\right)+\gamma H_{p}\left(Z\right). \end{array}$$
*The minimun is attained by $\left(R_{1},R_{2},R_{3}\right)=(0,H_{p}\left(Y\right),$$H_{p}\left(Z\right))$. This result implies that*
  $$\begin{array}{c} R\left(p_{XYZ}\right)\subseteq \begin{array}{c} \left[\underset{\gamma \in [0,1]}{⋂}\left\{\left(R_{1},R_{2},R_{3}\right):R_{1}+\bar{\gamma }R_{2}+\gamma R_{3}\geq \bar{\gamma }H_{p}\left(Y\right)+\gamma H_{p}\left(Z\right)\right\}\right]\cap R_{+}^{3}. \end{array} \end{array}$$
*Furthermore, the point $\left(0,H_{p}\left(Y\right),H_{p}\left(Z\right)\right)$ always belongs to $R(p_{XYZ})$.*

The rate region $R_{W}\left(p_{XYZ}\right)$ was determined by Wyner [2]. His result is the following.

**Theorem** **4** (Wyner [2]).

$$\begin{array}{c} R_{W}\left(p_{XYZ}\right)=R\left(p_{XYZ}\right). \end{array}$$

On the strong converse theorem, Csiszár and Körner [21] obtained the following.

**Theorem** **5** (Csiszár and Körner [21]). *For each fixed ε∈(0,1), we have*

$$\begin{array}{c} R_{W}\left(\varepsilon |p_{XYZ}\right)=R\left(p_{XYZ}\right). \end{array}$$

To examine a rate of convergence for the error probability of decoding to tend to one as n→∞ for $\left(R_{1},R_{2},R_{3}\right)$ $\notin R_{W}\left(p_{XYZ}\right)$, we define the following quantity. Set

Pc(n)(φ1(n),φ2(n),φ3(n),ψ(n),ϕ(n)):=1−Pe(n)(φ1(n),φ2(n),φ3(n),ψ(n),ϕ(n)),G(n)(R1,R2,R3|pXYZ):=min(φ1(n),φ2(n),φ3(n),ψ(n),ϕ(n)):(1/n)log∥φi(n)∥≤Ri,i=1,2,3−1nlogPc(n)(φ1(n),φ2(n),φ3(n),ψ(n),ϕ(n)),G(R1,R2,R3|pXYZ):=limn→∞G(n)(R1,R2,R3|pXYZ),G(pXYZ):={(R1,R2,R3,G):G≥G(R1,R2,R3|pXYZ)}.

By time sharing, we have that

(42) $$\begin{array}{c} G^{\left(n+m\right)}\left(\left.\frac{nR_{1}+mR_{1}^{\prime }}{n+m},\frac{nR_{2}+mR_{2}^{\prime }}{n+m},\frac{nR_{2}+mR_{2}^{\prime }}{n+m}\right|p_{XYZ}\right)\\ \leq \frac{nG^{\left(n\right)}\left(R_{1},R_{2},R_{3}|p_{XYZ}\right)+mG^{\left(m\right)}\left(R_{1}^{\prime },R_{2}^{\prime },R_{3}^{\prime }|p_{XYZ}\right)}{n+m}. \end{array}$$

Choosing $R=R^{\prime }$ in (42), we obtain the following subadditivity property on $\{G^{\left(n\right)}\left(R_{1},R_{2},R_{3}|p_{XYZ}\right)$ ${\}}_{n\geq 1}$:

$$\begin{array}{c} G^{\left(n+m\right)}\left(R_{1},R_{2},R_{3}|p_{XYZ}\right)\leq \frac{nG^{\left(n\right)}\left(R_{1},R_{2},R_{3}|p_{XYZ}\right)+mG^{\left(m\right)}\left(R_{1},R_{2},R_{3}|p_{XYZ}\right)}{n+m}, \end{array}$$

from which we have that $G(R_{1},R_{2},R_{3}|p_{XYZ})$ exists and satisfies the following:

$$\begin{array}{c} G\left(R_{1},R_{2},R_{3}|p_{XYZ}\right)=\underset{n\geq 1}{\inf}G^{\left(n\right)}\left(R_{1},R_{2},R_{3}|p_{XYZ}\right). \end{array}$$

The exponent function $G(R_{1},R_{2},R_{3}|p_{XYZ})$ is a convex function of $\left(R_{1},R_{2},R_{3}\right)$. In fact, by time sharing, we have that

$$\begin{array}{c} G^{\left(n+m\right)}\left(\left.\frac{nR_{1}+mR_{1}^{\prime }}{n+m},\frac{nR_{2}+mR_{2}^{\prime }}{n+m},\frac{nR_{2}+mR_{2}^{\prime }}{n+m}\right|p_{XYZ}\right)\\ \leq \frac{nG^{\left(n\right)}\left(R_{1},R_{2},R_{3}|p_{XYZ}\right)+mG^{\left(m\right)}\left(R_{1}^{\prime },R_{2}^{\prime },R_{3}^{\prime }|p_{XYZ}\right)}{n+m}, \end{array}$$

from which we have that for any α∈[0,1]

$$\begin{array}{c} G\left(\alpha R_{1}+\bar{\alpha }R_{1}^{\prime },\alpha R_{2}+\bar{\alpha }R_{2}^{\prime },\alpha R_{3}+\bar{\alpha }R_{3}^{\prime }|p_{XYZ}\right)\leq \alpha G\left(R_{1},R_{2},R_{3}|p_{XYZ}\right)+\bar{\alpha }G\left(R_{1}^{\prime },R_{2}^{\prime },R_{3}^{\prime }|p_{XYZ}\right). \end{array}$$

The region $G(p_{XYZ})$ is also a closed convex set. Our main aim is to find an explicit characterization of $G(p_{XYZ})$. In this paper, we derive an explicit outer bound of G $\left(p_{XYZ}\right)$ whose section by the plane G=0 coincides with $R_{W}\left(p_{XYZ}\right)$. We first explain that the region $R(p_{XYZ})$ has another expression using the supporting hyperplane. We define two sets of probability distributions on U ×X ×Y ×Z by

$$\begin{array}{c} P_{\mathrm{sh}}\left(p_{XYZ}\right):=\{p=p_{UXYZ}:|U|\leq |X|,U↔X↔YZ\},\\ Q\left(p_{YZ|X}\right):=\{q=q_{UXYZ}:|U|\leq |X|,p_{YZ|X}=q_{YZ|X},U↔X↔YZ\}. \end{array}$$

For $\left(\mu ,\gamma \right)\in {\left[0,1\right]}^{2}$, set

$$\begin{array}{c} R^{\left(\mu ,\gamma \right)}\left(p_{XYZ}\right):=\max_{p\in P_{\mathrm{sh}}\left(p_{XYZ}\right)}\left\{\mu I_{p}\left(X;U\right)+\bar{\mu }\left(\bar{\gamma }H_{p}\left(Y|U\right)+\gamma H_{p}\left(Z|U\right)\right)\right\}. \end{array}$$

Furthermore, define

$$\begin{array}{c} R_{\mathrm{sh}}\left(p_{XYZ}\right)=\underset{\left(\mu ,\gamma \right)\in {\left[0,1\right]}^{2}}{⋂}\left\{\left(R_{1},R_{2},R_{3}\right):\mu R_{1}+\bar{\mu }\left(\bar{\gamma }R_{2}+\gamma R_{3}\right)\geq R^{\left(\mu ,\gamma \right)}\left(p_{XYZ}\right)\right\}. \end{array}$$

Then, we have the following property.

**Property** **7.**

*(a)*  *The bound |U|≤|X| is sufficient to describe $R^{\left(\mu \right)}($$p_{XYZ})$.*
*(b)*  *For every $\left(\mu ,\gamma \right)\in {\left[0,1\right]}^{2}$, we have*
  $$\begin{array}{c} \min_{\left(R_{1},R_{2},R_{3}\right)\in R\left(p_{XYZ}\right)}\left\{\mu R_{1}+\bar{\mu }\left(\bar{\gamma }R_{2}+\gamma R_{3}\right)\right\}=R^{\left(\mu ,\gamma \right)}\left(p_{XYZ}\right). \end{array}$$
*(c)*  *For any $p_{XYZ},$ we have*
  (43) $$R_{\mathrm{sh}}\left(p_{XYZ}\right)=R\left(p_{XYZ}\right).$$

For $\left(\mu ,\gamma ,\alpha \right)\in {\left[0,1\right]}^{3}$, and for $q=q_{UXYZ}\in Q\left(p_{YZ|X}\right)$, define

ωq|pX(μ,γ,α)(x,y,z|u):=α¯logqX(x)pX(x)+αμlogqX|U(x|u)pX(x)+μ¯γ¯log1qY|U(y|u)+γlog1qZ|U(z|u),fq|pX(μ,γ,α)(x,y,z|u):=exp−ωq|pX(μ,γ,α)(x,y,z|u),Ω(μ,γ,α)(q|pX):=−logEqfq|pX(μ,γ,α)(X,Y,Z|U),Ω(μ,γ,α)(pXYZ):=minq∈Q(pYZ|X)Ω(μ,γ,α)(q|pX),F(μ,γ,α)(μR1+γ¯R2+γR3):=Ω(μ,γ,α)(pXYZ)−α[μR1+μ¯(γ¯R2+γR3)]2+αμ¯,F(R1,R2,R3|pXYZ):=sup(μ,γ,α)∈[0,1]3,F(μ,γ,α)(μR1+μ¯(γ¯R2+γR3)|pXYZ).

We next define a function serving as a lower bound of $F(R_{1},R_{2},R_{3}|p_{XYZ})$. For each $p=p_{UXYZ}\in P_{\mathrm{sh}}\left(p_{XYZ}\right)$, define

ω˜p(μ,γ)(x,y,z|u):=μlogpX|U(x|u)pX(x)+μ¯γ¯log1pY|U(y|u)+γlog1pZ|U(z|u),Ω˜(μ,γ,λ)(p):=−logEpexp−λωp(μ,γ)(X,Y,Z|U),Ω˜(μ,γ,λ)(pXYZ):=minp∈Psh(pXYZ)Ω˜(μ,γ,λ)(p).

Furthermore, set

F_(μ,γ,λ)(μR1+γ¯R2+γR3|pXYZ):=Ω˜(μ,γ,λ)(pXYZ)−λ[μR1+μ¯(γ¯R2+γR3)]2+λ(5−μ),F_(R1,R2,R3|pXYZ):=sup(μ,γ)∈[0,1]2,λ≥0F_(μ,γ,λ)(μR1+μ¯γ¯R2+γR3|pXYZ).

We can show that the above functions and sets satisfy the following property.

**Property** **8.**

*(a)*  *The cardinality bound |U|≤|X| in $Q(p_{Y|X})$ is sufficient to describe the quantity $\Omega^{\left(\mu ,\alpha \right)}\left(p_{XY}\right)$. Furthermore, the cardinality bound |U|≤|X| in $Q(p_{YZ|X})$ is sufficient to describe the quantity ${\tilde{\Omega }}^{\left(\mu ,\gamma ,\lambda \right)}\left(p_{XYZ}\right)$.*
*(b)*  *For any $R_{1},R_{2},R_{3}\geq 0$, we have*
  $$\begin{array}{c} F\left(R_{1},R_{2},R_{3}|p_{XYZ}\right)\geq \underline{F}\left(R_{1},R_{2},R_{3}|p_{XYZ}\right). \end{array}$$
*(c)*  *For any $p=p_{UXY}\in P_{\mathrm{sh}}\left(p_{XY}\right)$ and any (μ,γ,λ)∈[0,${1]}^{3}$, we have*
  (44) $$0\leq {\tilde{\Omega }}^{\left(\mu ,\gamma ,\lambda \right)}\left(p\right)\leq \mu \log|X|+\bar{\mu }{\log(|Y|}^{\bar{\gamma }}{\left|Z\right|}^{\gamma }).$$
*(d)*  *Fix any $p=p_{UXYZ}\in P_{\mathrm{sh}}\left(p_{XYZ}\right)$ and $\left(\mu ,\gamma \right)\in {\left[0,1\right]}^{2}$. We define a probability distribution $p^{\left(\lambda \right)}=p_{UXYZ}^{\left(\lambda \right)}$ by*
  $$\begin{array}{c} p^{\left(\lambda \right)}\left(u,x,y,z\right):=\frac{p\left(u,x,y,z\right)\exp\left\{-\lambda \omega_{p}^{\left(\mu ,\gamma \right)}\left(x,y,z|u\right)\right\}}{E_{p}\left[\exp\left\{-\lambda \omega_{p}^{\left(\mu ,\gamma \right)}\left(X,Y,Z|U\right)\right\}\right]}. \end{array}$$
*Then, for λ∈[0,1/2], ${\tilde{\Omega }}^{\left(\mu ,\gamma ,\lambda \right)}\left(p\right)$ is twice differentiable. Furthermore, for λ∈[0,1/2], we have*
  $$\begin{array}{c} \frac{d}{d\lambda }{\tilde{\Omega }}^{\left(\mu ,\gamma ,\lambda \right)}\left(p\right)=E_{p^{\left(\lambda \right)}}\left[\omega_{p}^{\left(\mu ,\gamma \right)}\left(X,Y,Z|U\right)\right],\frac{d^{2}}{d\lambda^{2}}{\tilde{\Omega }}^{\left(\mu ,\gamma ,\lambda \right)}\left(p\right)=-{\mathrm{Var}}_{p^{\left(\lambda \right)}}\left[\omega_{p}^{\left(\mu ,\gamma \right)}\left(X,Y,Z|U\right)\right]. \end{array}$$
*The second equality implies that ${\tilde{\Omega }}^{\left(\mu ,\gamma ,\lambda \right)}\left(p\right)$ is a concave function of λ∈[0,1/2].*
*(e)*  *For $\left(\mu ,\gamma ,\lambda \right)\in {\left[0,1\right]}^{2}\times \left[0,1/2\right]$, define*
  ρ(μ,γ,λ)(pXYZ):=max(ν,p)∈[0,λ]×Psh(pXYZ):Ω˜(μ,γ,λ)(p)=Ω˜(μ,γ,λ)(pXYZ)Varp(ν)ω˜p(μ,γ)(X,Y,Z|U),
  *and set*
  $$\begin{array}{c} \rho =\rho \left(p_{XYZ}\right):=\max_{\left(\mu ,\gamma ,\lambda \right)\in {\left[0,1\right]}^{2}\times \left[0,1/2\right]}\rho^{\left(\mu ,\gamma ,\lambda \right)}\left(p_{XYZ}\right). \end{array}$$
*Then, we have $\rho (p_{XYZ})<\infty$. Furthermore, for any (μ,γ,λ)$\in {\left[0,1\right]}^{2}\times \left[0,1/2\right]$, we have*
  $${\tilde{\Omega }}^{\left(\mu ,\gamma ,\lambda \right)}\left(p_{XYZ}\right)\geq \lambda R^{\left(\mu ,\gamma \right)}\left(p_{XYZ}\right)-\frac{\lambda^{2}}{2}\rho \left(p_{XYZ}\right).$$
*(f)*  *For every $\tau \in (0,\left(1/2\right)\rho \left(p_{XYZ}\right))$, the condition $(R_{1}+\tau ,$$R_{2}+\tau ,R_{3}+\tau )\notin R\left(p_{XYZ}\right)$ implies*
  $$\begin{array}{c} F\left(R_{1},R_{2},R_{3}|p_{XYZ}\right)>\frac{\rho (p_{XYZ})}{4}\cdot g^{2}\left(\frac{\tau }{\rho (p_{XYZ})}\right)>0. \end{array}$$

Since proofs of the results stated in Property 8 are quite parallel with those of the results stated in Property 4, we omit them. Our main result is the following.

**Theorem** **6.** *For any $R_{1},R_{2},$$R_{3}\geq 0$, any $p_{XYZ}$, and for any $(\varphi_{1}^{\left(n\right)},$$\varphi_{2}^{\left(n\right)},$$\varphi_{3}^{\left(n\right)},$$\psi^{\left(n\right)},\phi^{\left(n\right)})$ satisfying $\left(1/n\right)\log||\varphi_{i}^{\left(n\right)}\left|\right|$$\leq R_{i},i=1,2,3,$ we have*

$$\begin{array}{c} P_{c}^{\left(n\right)}\left(\varphi_{1}^{\left(n\right)},\varphi_{2}^{\left(n\right)},\varphi_{3}^{\left(n\right)},\psi^{\left(n\right)},\phi^{\left(n\right)}\right)\leq 7\exp\left\{-nF(R_{1},R_{2},R_{3}|p_{XYZ})\right\}. \end{array}$$

It follows from Theorem 6 and Property 8 part d) that, if $\left(R_{1},R_{2},R_{3}\right)$ is outside the capacity region, then the error probability of decoding goes to one exponentially and its exponent is not below $F(R_{1},R_{2},R_{3}|p_{XYZ})$. It immediately follows from Theorem 3 that we have the following corollary.

**Corollary** **4.** $$\begin{array}{c} G\left(R_{1},R_{2},R_{3}|p_{XYZ}\right)\geq F\left(R_{1},R_{2},R_{3}|p_{XYZ}\right),\\ G\left(p_{XYZ}\right)\subseteq \bar{G}\left(p_{XYZ}\right)=\left\{\left(R_{1},R_{2},R_{3},G\right):G\geq F\left(R_{1},R_{2},R_{3}|p_{XYZ}\right)\right\}. \end{array}$$

Proof of Theorem 6 is quite parallel with that of Theorem 3. We omit the detail of the proof. From Theorem 6 and Property 8 part e, we can obtain an explicit outer bound of $R_{W}\left(\varepsilon |p_{XYZ}\right)$ with an asymptotically vanishing deviation from $R_{W}\left(p_{XYZ}\right)$$=R(p_{XYZ})$. The strong converse theorem established by Csiszár and Körner [21] immediately follows from this corollary. To describe this outer bound, for κ>0, we set

$$\begin{array}{c} R\left(p_{XYZ}\right)-\kappa \left(1,1,1\right):=\left\{\left(R_{1}-\kappa ,R_{2}-\kappa ,R_{3}-\kappa \right):\left(R_{1},R_{2},R_{3}\right)\in R\left(p_{XYZ}\right)\right\}, \end{array}$$

which serves as an outer bound of $R(p_{XYZ})$. For each fixed ε∈(0,1), we define ${\tilde{\kappa }}_{n}$$={\tilde{\kappa }}_{n}\left(\varepsilon ,\rho \left(p_{XYZ}\right)\right)$ by

(45) $$\begin{array}{ccc} {\tilde{\kappa }}_{n} & := & \rho \left(p_{XY}\right)\vartheta \left(\sqrt{\frac{4}{n\rho (p_{XY})}\log\left(\frac{7}{1-\varepsilon }\right)}\right)\\ & \overset{\left(a\right)}{=} & 2\sqrt{\frac{\rho (p_{XY})}{n}\log\left(\frac{7}{1-\varepsilon }\right)}+\frac{5}{n}\log\left(\frac{7}{1-\varepsilon }\right). \end{array}$$

Step (a) follows from $\vartheta \left(a\right)=a+\left(5/4\right)a^{2}$. Since ${\tilde{\kappa }}_{n}\to 0$ as n→∞, we have the smallest positive integer $n_{1}=n_{1}\left(\varepsilon ,\rho \left(p_{XYZ}\right)\right)$ such that ${\tilde{\kappa }}_{n}\leq \left(1/2\right)\rho \left(p_{XYZ}\right)$ for $n\geq n_{1}$. From Theorem 6 and Property 8 part e, we have the following corollary.

**Corollary** **5.** *For each fixed ε∈(0,1), we choose the above positive integer $n_{1}=$$n_{1}\left(\varepsilon ,\rho \left(p_{XYZ}\right)\right)$. Then, for any $n\geq n_{1}$, we have*

$$\begin{array}{cc} R_{W}\left(\varepsilon |p_{XYZ}\right) & \subseteq R\left(p_{XYZ}\right)-{\tilde{\kappa }}_{n}\left(0,1,1\right). \end{array}$$

*The above result together with*

$$\begin{array}{cc} R_{W}\left(\varepsilon |p_{XYZ}\right) & =\mathrm{cl}\left(\underset{m\geq 1}{⋃}\underset{n\geq m}{⋂}R_{W}\left(n,\varepsilon |p_{XYZ}\right)\right) \end{array}$$

*yields that for each fixed ε∈(0,1), we have*

$$\begin{array}{c} R_{W}\left(\varepsilon |p_{XYZ}\right)=R_{W}\left(p_{XYZ}\right)=R\left(p_{XYZ}\right). \end{array}$$

*This recovers the strong converse theorem proved by Csiszár and Körner [21].*

Proof of this corollary is quite parallel with that of Corollary 2. We omit the detail.

## 6. Conclusions

For the AWZ system, the one helper source coding system posed by Ahlswede, Körner [1] and Wyner [2], we have derived an explicit lower bound of the optimal exponent function $G(R_{1},R_{2}|p_{XY})$ on the correct probability of decoding for $\left(R_{1},R_{2}\right)\notin R_{\mathrm{WZ}}\left(p_{XY}\right)$. We have described this result in Theorem 3. Furthermore, for the source coding system posed and investigated Wyner [2], we have obtained an explicit lower bound of the optimal exponent function $G(R_{1},R_{2},R_{3}|p_{XYZ})$ on the correct probability of decoding for $\left(R_{1},R_{2},R_{3}\right)\notin R_{W}\left(p_{XYZ}\right)$. We have described this result in Theorem 6. The determination problems of $G(R_{1},R_{2}|p_{XY})$ and $G(R_{1},R_{2},R_{3}|p_{XYZ})$ still remain to be resolved. Those problems are our future works.

## Appendix A. Properties of the Rate Regions

In this appendix, we prove Property 1. Property 1 part a can easily be proved by the definitions of the rate distortion regions. We omit the proofs of this part. In the following argument, we prove the part b.

**Proof** **of** **Property** **1 part b:** We set

$$\begin{array}{cc} {\underline{R}}_{\mathrm{AKW}}\left(m,\varepsilon |p_{XY}\right) & =\underset{n\geq m}{⋂}R_{\mathrm{AKW}}\left(n,\varepsilon |p_{XY}\right). \end{array}$$

By the definitions of ${\underline{R}}_{\mathrm{AKW}}\left(m,\varepsilon |p_{XY}\right)$ and $R_{\mathrm{AKW}}\left(\varepsilon |p_{XY}\right)$, we have that ${\underline{R}}_{\mathrm{AKW}}\left(m,\varepsilon |p_{XY}\right)$ $\subseteq R_{\mathrm{AKW}}\left(\varepsilon |p_{XY}\right)$ for m≥1. Hence, we have that

(A1) $$\underset{m\geq 1}{⋃}{\underline{R}}_{\mathrm{AKW}}\left(m,\varepsilon |p_{XY}\right)\subseteq R_{\mathrm{AKW}}\left(\varepsilon |p_{XY}\right).$$

We next assume that $\left(R_{1},R_{2}\right)\in R_{\mathrm{AKW}}\left(\varepsilon |p_{XY}\right)$. Set

$$\begin{array}{c} R_{\mathrm{AKW}}^{\left(\delta \right)}\left(\varepsilon |p_{XY}\right):=\left\{\left(R_{1}+\delta ,R_{2}+\delta \right):\left(R_{1},R_{2}\right)\in R_{\mathrm{AKW}}\left(\varepsilon |p_{XY}\right)\right\}. \end{array}$$

Then, by the definitions of $R_{\mathrm{AKW}}(n,\varepsilon$$|p_{XY})$ and $R_{\mathrm{AKW}}($$\varepsilon |p_{XY})$, we have that, for any δ>0, there exists $n_{0}\left(\varepsilon ,\delta \right)$ such that for any $n\geq n_{0}\left(\varepsilon ,\delta \right)$, $\left(R_{1}+\delta ,R_{2}+\delta \right)\in R_{\mathrm{AKW}}\left(n,\varepsilon |p_{XY}\right),$ which implies that

(A2) $$\begin{array}{ccc} R_{\mathrm{AKW}}^{\left(\delta \right)}\left(\varepsilon |p_{XY}\right) & \subseteq & \underset{n\geq n_{0}\left(\varepsilon ,\delta \right)}{⋂}R_{\mathrm{AKW}}\left(n,\varepsilon |p_{XY}\right)={\underline{R}}_{\mathrm{AKW}}\left(n_{0}\left(\varepsilon ,\delta \right),\varepsilon |p_{XY}\right)\\ & \subseteq & \mathrm{cl}\left(\underset{m\geq 1}{⋃}{\underline{R}}_{\mathrm{AKW}}\left(m,\varepsilon |p_{XY}\right)\right). \end{array}$$

Here, we assume that there exists a pair $\left(R_{1},R_{2}\right)$ belonging to $R_{\mathrm{AKW}}\left(\varepsilon |p_{XY}\right)$ such that

(A3) $$\left(R_{1},R_{2}\right)\notin \mathrm{cl}\left(\underset{m\geq 1}{⋃}{\underline{R}}_{\mathrm{AKW}}\left(m,\varepsilon |p_{XY}\right)\right).$$

Since the set on the right-hand side of (A3) is a closed set, we have

(A4) $$\left(R_{1}+\delta ,R_{2}+\delta \right)\notin \mathrm{cl}\left(\underset{m\geq 1}{⋃}{\underline{R}}_{\mathrm{AKW}}\left(m,\varepsilon |p_{XY}\right)\right)$$

for some small δ>0. On the other hand, we have $\left(R_{1}+\delta ,R_{2}+\delta \right)$ $\in R_{\mathrm{AKW}}^{\left(\delta \right)}\left(\varepsilon |p_{XY}\right)$, which contradicts (A2). Thus, we have

(A5) $$\begin{array}{c} \underset{m\geq 1}{⋃}{\underline{R}}_{\mathrm{AKW}}\left(m,\varepsilon |p_{XY}\right)\subseteq R_{\mathrm{AKW}}\left(\varepsilon |p_{XY}\right)\subseteq \mathrm{cl}\left(\underset{m\geq 1}{⋃}{\underline{R}}_{\mathrm{AKW}}\left(m,\varepsilon |p_{XY}\right)\right).\; \end{array}$$

Note here that $R_{\mathrm{AKW}}$(ε$|p_{XY})$ is a closed set. Then, from (A5), we conclude that

$$\begin{array}{cc} R_{\mathrm{AKW}}\left(\varepsilon |W\right) & =\mathrm{cl}\left(\underset{m\geq 1}{⋃}{\underline{R}}_{\mathrm{AKW}}\left(m,\varepsilon |p_{XY}\right)\right)=\mathrm{cl}\left(\underset{m\geq 1}{⋃}\underset{n\geq m}{⋂}R_{\mathrm{AKW}}\left(n,\varepsilon |p_{XY}\right)\right), \end{array}$$

completing the proof. □

## Appendix B. Cardinality Bound on Auxiliary Random Variables

We first prove the following lemma.

**Lemma** **A1.**

$$\begin{array}{c} {\underline{R}}^{\left(\mu \right)}\left(p_{XY}\right):=\min_{p\in P(p_{XY})}\left\{\mu I_{p}\left(X;U\right)+\bar{\mu }H_{p}\left(Y|U\right)\right\}\\ =R^{\left(\mu \right)}\left(p_{XY}\right):=\min_{p\in P_{\mathrm{sh}}\left(p_{XY}\right)}\left\{\mu I_{p}\left(X;U\right)+\bar{\mu }H_{p}\left(Y|U\right)\right\}. \end{array}$$

**Proof.** We bound the cardinality |U| of *U* to show that the bound |U|≤|X| is sufficient to describe ${\underline{R}}^{\left(\mu \right)}\left(p_{XY}\right)$. Observe that

(A6) $$\begin{array}{c} p_{X}\left(x\right)=\sum_{u\in U}p_{U}\left(u\right)p_{X|U}\left(x|u\right), \end{array}$$

(A7) $$\begin{array}{c} \mu I_{p}\left(X;U\right)+\bar{\mu }H_{p}\left(Y|U\right)=\sum_{u\in U}p_{U}\left(u\right)\pi \left(p_{X|U}\left(\cdot |u\right)\right), \end{array}$$

where

$$\begin{array}{c} \pi \left(p_{X|U}\left(\cdot |u\right)\right):=\sum_{(x,y)\in X\times Y}p_{X|U}\left(x|u\right)p_{Y|X}\left(y|x\right)\log\left\{\frac{p_{X|U}^{\mu }\left(x|u\right)}{p_{X}^{\mu }\left(x\right)}{\left[\sum_{\tilde{x}\in X}p_{Y|X}\left(y|\tilde{x}\right)p_{X|U}\left(\tilde{x}|u\right)\right]}^{-\bar{\mu }}\right\}. \end{array}$$

For each u∈U, $\pi (p_{X|U}\left(\cdot |u\right))$ is a continuous function of $p_{X|U}\left(\cdot |u\right)$. Then, by the support lemma, |U|≤|X|−1+1=|X| is sufficient to express |X|−1 values of (A6) and one value of (A7). □

Next, we prove the following lemma.

**Lemma** **A2.**
*The cardinality bound |U|≤|X| in $Q(p_{Y|X})$ is sufficient to describe the quantity $\Omega^{\left(\mu ,\alpha \right)}\left(p_{XY}\right)$. The cardinality bound |U|≤|X| in $P_{\mathrm{sh}}\left(p_{XY}\right)$ is sufficient to describe the quantity ${\tilde{\Omega }}^{\left(\mu ,\lambda \right)}\left(p_{XY}\right)$.*

**Proof.** We first bound the cardinality |U| of *U* in $Q(p_{Y|X})$ to show that the bound |U|≤|X| is sufficient to describe $\Omega^{\left(\mu ,\alpha \right)}$$\left(p_{XY}\right)$. Observe that

(A8) $$\begin{array}{c} q_{X}\left(x\right)=\sum_{u\in U}q_{U}\left(u\right)q_{X|U}\left(x|u\right), \end{array}$$

(A9) $$\begin{array}{c} \exp\left\{-\Omega^{\left(\mu ,\alpha \right)}\left(q|p_{X}\right)\right\}=\sum_{u\in U}q_{U}\left(u\right)\Pi^{\left(\mu ,\alpha \right)}\left(q_{X},q_{XY|U}\left(\cdot ,\cdot |u\right)\right), \end{array}$$

where

Π(μ,α)(qX,qXY|U(·,·|u)):=∑(x,y)∈X×YqXY|U(x,y|u)exp−ωq|pX(μ,α)(x,y|u).

The value of $q_{X}$ included in $\Pi^{\left(\mu ,\alpha \right)}\left(q_{X},q_{XY|U}\left(\cdot ,\cdot |u\right)\right)$ must be preserved under the reduction of U. For each u∈U, $\Pi^{\left(\mu ,\alpha \right)}\left(q_{X},q_{XY|U}\left(\cdot ,\cdot |u\right)\right)$ is a continuous function of $q_{XY|U}($·,·|u). Then, by the support lemma, |U|≤|X|−1+1=|X| is sufficient to express |X|−1 values of (A8) and one value of (A9). We next bound the cardinality |U| of *U* in $P_{\mathrm{sh}}\left(p_{XY}\right)$ to show that the bound |U|≤|X| is sufficient to describe ${\tilde{\Omega }}^{\left(\mu ,\lambda \right)}$$\left(p_{XY}\right)$. Observe that

(A10) $$\begin{array}{c} p_{X}\left(x\right)=\sum_{u\in U}p_{U}\left(u\right)p_{X|U}\left(x|u\right), \end{array}$$

(A11) $$\begin{array}{c} \exp\left\{-{\tilde{\Omega }}^{\left(\mu ,\lambda \right)}\left(p\right)\right\}=\sum_{u\in U}p_{U}\left(u\right){\tilde{\Pi }}^{\left(\mu ,\lambda \right)}\left(p_{X},p_{XY|U}\left(\cdot ,\cdot |u\right)\right), \end{array}$$

where

Π˜(μ,λ)(pX,pXY|U(·,·|u)):=∑(x,y)∈X×YpXY|U(x,y|u)exp−λω˜p(μ)(x,y|u).

The value of $p_{X}$ included in ${\tilde{\Pi }}^{\left(\mu ,\lambda \right)}\left(p_{X},p_{XY|U}\left(\cdot ,\cdot |u\right)\right)$ must be preserved under the reduction of U. For each u∈U, ${\tilde{\Pi }}^{\left(\mu ,\lambda \right)}\left(p_{X},p_{XY|U}\left(\cdot ,\cdot |u\right)\right)$ is a continuous function of $p_{XY|U}\left(\cdot ,\cdot |u\right)$. Then, by the support lemma, |U|≤|X|−1+1=|X| is sufficient to express |X|−1 values of (A10) and one value of (A11). □

## Appendix C. Supporting Hyperplain Expressions of $R(p_{XY})$

In this appendix we prove Property 3 parts (b), (c). We first prove the part (b).

**Proof** **of** **Property** **3** **part** **b:** For any μ≥0, we have the following chain of inequalities:

$$\begin{array}{c} \min_{\left(R_{1},R_{2}\right)\in R\left(p_{XY}\right)}\left\{\mu R_{1}+\bar{\mu }R_{2}\right\}\\ =\min_{p\in P_{}\left(p_{XY}\right)}\left\{\mu I_{p}\left(X;U\right)+\bar{\mu }H_{p}\left(Y|U\right)\right\}\overset{\left(a\right)}{=}\min_{p\in P_{\mathrm{sh}}\left(p_{XY}\right)}\left\{\mu I_{p}\left(X;U\right)+\bar{\mu }H_{p}\left(Y|U\right)\right\}=R^{\left(\mu \right)}\left(p_{XY}\right). \end{array}$$

Step (a) follows from Lemma A1 stating that the cardinality bound |U|≤|X|+1 in $P_{}\left(p_{XY}\right)$ can be reduced to that |U|≤|X| in $P_{\mathrm{sh}}\left(p_{XY}\right)$. □

We next prove part c. We first prepare a lemma useful to prove this property. From the convex property of the region $R(p_{XY})$, we have the following lemma.

**Lemma** **A3.**
*Suppose that $\left({\hat{R}}_{1},{\hat{R}}_{2}\right)$ does not belong to $R(p_{XY})$. Then, there exist ϵ>0 and $\mu_{0}\geq 0$ such that for any $\left(R_{1},R_{2}\right)\in R\left(p_{XY}\right)$ we have*

$$\begin{array}{c} \mu_{0}\left(R_{1}-{\hat{R}}_{1}\right)+\bar{\mu_{0}}\left(R_{2}-{\hat{R}}_{2}\right)-\epsilon \geq 0. \end{array}$$

Proof of this lemma is omitted here. Lemma A3 is equivalent to the fact that if the region $R(p_{XY})$ is a convex set; then, for any point $\left({\hat{R}}_{1},{\hat{R}}_{2}\right)$ outside the region $R(p_{XY})$, there exists a line which separates the point $\left({\hat{R}}_{1},{\hat{R}}_{2}\right)$ from the region $R(p_{XY})$.

**Proof** **of** **Property** **3** **part** **c:** We first prove ${\underline{R}}_{\mathrm{sh}}\left(p_{XY}\right)$ $\subseteq R(p_{XY})$. We assume that $\left({\hat{R}}_{1},{\hat{R}}_{2}\right)\notin R\left(p_{XY}\right)$. Then, by Lemma A3, there exist ϵ>0 and $\mu_{0}\geq 0$ such that for any $\left(R_{1},R_{2}\right)\in R\left(p_{XY}\right)$, we have

$$\begin{array}{c} \mu_{0}{\hat{R}}_{1}+\bar{\mu_{0}}{\hat{R}}_{2}\leq \mu_{0}R_{1}+\bar{\mu_{0}}R_{2}-\epsilon . \end{array}$$

Then, we have

(A12) $$\begin{array}{c} \mu_{0}{\hat{R}}_{1}+\bar{\mu_{0}}{\hat{R}}_{2}\leq \min_{\left(R_{1},R_{2}\right)\in R\left(p_{XY}\right)}\left\{\mu_{0}R_{1}+\bar{\mu_{0}}R_{2}\right\}-\epsilon \overset{\left(a\right)}{=}\min_{p\in P(p_{XY})}\left\{\mu_{0}I_{p}\left(U;X\right)+\bar{\mu_{0}}H_{p}\left(Y|U\right)\right\}-\epsilon \\ \leq \min_{p\in P_{\mathrm{sh}}\left(p_{XY}\right)}\left\{\mu_{0}I_{p}\left(U;X\right)+\bar{\mu_{0}}H_{p}\left(Y|U\right)\right\}-\epsilon =R^{\left(\mu_{0}\right)}\left(p_{XY}\right)-\epsilon . \end{array}$$

Step (a) follows from the definition of $R(p_{XY})$. The inequality (A12) implies that $\left({\hat{R}}_{1},{\hat{R}}_{2}\right)$$\notin R_{\mathrm{sh}}\left(p_{XY}\right)$. Thus $R_{\mathrm{sh}}\left(p_{XY}\right)$$\subseteq R(p_{XY})$ is concluded. □

## Appendix D. Proof of Property 4 Part b

In this appendix, we prove Property 4 part b. Fix $q=q_{UXY}\in Q\left(p_{Y|X}\right)$ and $p=p_{UXY}=\left(p_{U|X},p_{XY}\right)\in P_{\mathrm{sh}}($$p_{XY})$ arbitrary. For β≥0, $p\in P_{\mathrm{sh}}\left(p_{XY}\right)$, and $q_{Y|U}$ induced by *q*, define

$$\begin{array}{c} {\hat{\omega }}_{p,q_{Y|U}}^{\left(\mu \right)}\left(x,y|u\right):=\mu \log\frac{p_{X|U}\left(x|u\right)}{p_{X}\left(x\right)}+\bar{\mu }\log\frac{1}{q_{Y|U}\left(y|u\right)},\\ {\hat{\Omega }}^{\left(\mu ,\beta \right)}\left(p,q_{Y|U}\right):=-\log E_{p}\left[\exp\left\{-\beta {\hat{\omega }}_{p,q_{Y|U}}^{\left(\mu \right)}\left(X,Y|U\right)\right\}\right]. \end{array}$$

Then, we have the following two lemmas.

**Lemma** **A4.**
*For any μ∈[0,1], α∈[0,1), and any $q=q_{UXY}\in Q\left(p_{Y|X}\right)$, there exists $p=p_{UXY}\in P_{\mathrm{sh}}\left(p_{XY}\right)$ such that*

(A13) $$\Omega^{\left(\mu ,\alpha \right)}\left(q|p_{X}\right)\geq \bar{\alpha }{\hat{\Omega }}^{\left(\mu ,\frac{\alpha }{\bar{\alpha }}\right)}\left(p,q_{Y|U}\right).$$

**Lemma** **A5.**
*For any μ,α satisfying μ∈[0,1], α∈[0,1/2), any $p=p_{UXY}\in P_{\mathrm{sh}}\left(p_{XY}\right)$, and any stochastic matrix $q_{Y|U}$ induced by $q_{UXY}\in Q\left(p_{Y|X}\right)$, we have*

(A14) $$\begin{array}{c} {\hat{\Omega }}^{\left(\mu ,\frac{\alpha }{\bar{\alpha }}\right)}\left(p,q_{Y|U}\right)\geq \frac{1-2\alpha }{\bar{\alpha }}{\tilde{\Omega }}^{\left(\mu ,\frac{\alpha }{1-2\alpha }\right)}\left(p\right). \end{array}$$

From Lemmas A4 and A5, we have the following corollary.

**Corollary** **A1.**
*For any μ,α satisfying μ∈[0,1], α∈[0,1/2), and any $q=q_{UXY}\in Q\left(p_{Y|X}\right)$, there exists $p=p_{UXY}\in P_{\mathrm{sh}}\left(p_{XY}\right)$ such that*

(A15) $$\begin{array}{c} \Omega^{\left(\mu ,\alpha \right)}\left(q|p_{X}\right)\geq \left(1-2\alpha \right){\tilde{\Omega }}^{\left(\mu ,\frac{\alpha }{1-2\alpha }\right)}\left(p\right). \end{array}$$

*From (A15), we have that for any μ∈[0,1], α∈[0,1/2), we have*

(A16) $$\begin{array}{c} \Omega^{\left(\mu ,\alpha \right)}\left(p_{XY}\right)\geq \left(1-2\alpha \right){\tilde{\Omega }}^{\left(\mu ,\frac{\alpha }{1-2\alpha }\right)}\left(p_{XY}\right). \end{array}$$

**Proof** **of** **Lemma** **A4:** We fix $\left(\mu ,\alpha \right)\in {\left[0,1\right]}^{2}$ arbitrary. For each $q=q_{UXY}\in Q\left(p_{Y|X}\right)$, we choose $p=p_{UXY}\in P_{\mathrm{sh}}\left(p_{XY}\right)$ so that $p_{U|X}=q_{U|X}$. Then, we have the following:

(A17) $$\begin{array}{c} \exp\left\{-\Omega^{\left(\mu ,\alpha \right)}\left(q|p_{X}\right)\right\}=E_{q}\left[\frac{p_{X}^{\bar{\alpha }}\left(X\right)}{q_{X}^{\bar{\alpha }}\left(X\right)}\left\{\frac{p_{X}^{\mu \alpha }\left(X\right)q_{Y|U}^{\bar{\mu }\alpha }\left(Y|U\right)}{q_{X|U}^{\mu \alpha }\left(X|U\right)}\right\}\right]\\ =E_{q}\left[{\left\{\frac{p_{UX}\left(U,X\right)}{q_{UX}\left(U,X\right)}\right\}}^{\bar{\alpha }}{\left\{\frac{p_{X}^{\mu \frac{\alpha }{\bar{\alpha }}}\left(X\right)q_{Y|U}^{\bar{\mu }\frac{\alpha }{\bar{\alpha }}}\left(Y|U\right)}{p_{X|U}^{\mu \frac{\alpha }{\bar{\alpha }}}\left(X|U\right)}\right\}}^{\bar{\alpha }}{\left\{\frac{p_{X|U}^{\mu }\left(X|U\right)}{q_{X|U}^{\mu }\left(X|U\right)}\right\}}^{\alpha }\right]\\ \overset{\left(a\right)}{\leq }{\left(E_{q}\left[\frac{p_{UX}\left(U,X\right)}{q_{UX}\left(U,X\right)}\frac{p_{X}^{\mu \frac{\alpha }{\bar{\alpha }}}\left(X\right)q_{Y|U}^{\bar{\mu }\frac{\alpha }{\bar{\alpha }}}\left(Y|U\right)}{p_{X|U}^{\mu \frac{\alpha }{\bar{\alpha }}}\left(X|U\right)}\right]\right)}^{\bar{\alpha }}{\left(E_{q}\left[\frac{p_{X|U}^{\mu }\left(X|U\right)}{q_{X|U}^{\mu }\left(X|U\right)}\right]\right)}^{\alpha }\\ =\exp\left\{-\bar{\alpha }{\hat{\Omega }}^{\left(\mu ,\frac{\alpha }{\bar{\alpha }}\right)}\left(p,q_{Y|U}\right)\right\}A^{\alpha }, \end{array}$$

where we set

$$A:=E_{q}\left[\frac{p_{X|U}^{\mu }\left(X|U\right)}{q_{X|U}^{\mu }\left(X|U\right)}\right].$$

Step (a) follows from Hölder’s inequality. From (A17), we can see that it suffices to show A≤1 to complete the proof. When μ=1, we have A=1. When μ∈[0,1), we apply Hölder’s inequality to *A* to obtain

$$\begin{array}{cc} A & =E_{q}\left[\frac{p_{X|U}^{\mu }\left(X|U\right)}{q_{X|U}^{\mu }\left(X|U\right)}\right]\leq {\left(E_{q}\left[\frac{p_{X|U}\left(X|U\right)}{q_{X|U}\left(X|U\right)}\right]\right)}^{\mu }=1. \end{array}$$

Hence, we have (A13) in Lemma A4. □

**Proof** **of** **Lemma** **A5:** We fix μ∈[0,1], α∈[0,1/2), arbitrary. For any $p=p_{UXY}\in P_{\mathrm{sh}}\left(p_{XY}\right)$, and any $q=q_{UXY}\in Q\left(p_{Y|X}\right)$, we have the following chain of inequalities:

(A18) $$\begin{array}{c} \exp\left\{-{\hat{\Omega }}^{\left(\mu ,\frac{\alpha }{\bar{\alpha }}\right)}\left(p,q_{Y|U}\right)\right\}=E_{p}\left[\begin{array}{c} \;\\ \;\\ \;\;\\ \; \end{array}\right.\;\;{\left\{\frac{p_{X}^{\mu \frac{\alpha }{1-2\alpha }}\left(X\right)p_{Y|U}^{\bar{\mu }\frac{\alpha }{1-2\alpha }}\left(Y|U\right)}{p_{X|U}^{\mu \frac{\alpha }{1-2\alpha }}\left(X|U\right)}\right\}}^{\frac{1-2\alpha }{\bar{\alpha }}}{\left\{\frac{q_{Y|U}^{\bar{\mu }}\left(Y|U\right)}{p_{Y|U}^{\bar{\mu }}\left(Y|U\right)}\right\}}^{\frac{\alpha }{\bar{\alpha }}}\left.\begin{array}{c} \;\\ \;\\ \;\;\\ \; \end{array}\;\;\right]\\ \overset{\left(a\right)}{\leq }\exp\left\{-\frac{1-2\alpha }{\bar{\alpha }}{\tilde{\Omega }}^{\left(\mu ,\frac{\alpha }{1-2\alpha }\right)}\left(p\right)\right\}{\left(E_{p}\left[\frac{q_{Y|U}^{\bar{\mu }}\left(Y|U\right)}{p_{Y|U}^{\bar{\mu }}\left(Y|U\right)}\right]\right)}^{\frac{\alpha }{\bar{\alpha }}}=\exp\left\{-\frac{1-2\alpha }{\bar{\alpha }}{\tilde{\Omega }}^{\left(\mu ,\frac{\alpha }{1-2\alpha }\right)}\left(p\right)\right\}B^{\frac{\alpha }{\bar{\alpha }}}, \end{array}$$

where we set

$$B:=E_{q}\left[\frac{q_{Y|U}^{\bar{\mu }}\left(Y|U\right)}{p_{Y|U}^{\bar{\mu }}\left(Y|U\right)}\right].$$

Step (a) follows from Hölder’s inequality. From (A18), we can see that it suffices to show B≤1 to complete the proof. In a manner quite smilar to the proof of A≤1 in the proof of (A13) in Lemma A4, we can show that B≤1. Thus, we have (A14) in Lemma A5. □

**Proof** **of** **Property** **4** **part** **b:** We evaluate lower bounds of $F(R_{1},R_{2}|p_{XY})$ to obtain the following chain of inequalities:

(A19) F(R1,R2|pXY)≥(a)supμ∈[0,1],α∈[0,1/2)(1−2α)Ω˜(μ,α1−2α)(pXY)−α(μR1+μ¯R2)2+αμ¯=supμ∈[0,1],α∈[0,1/2),λ=α1−2α(1−2α)Ω˜(μ,λ)(pXY)−α(μR1+μ¯R2)2+αμ¯=(b)supμ∈[0,1],α=λ1+2λ,λ≥0(1−2α)Ω˜(μ,λ)(pXY)−α(μR1+μ¯R2)2+αμ¯=(c)supμ∈[0,1],λ≥0Ω˜(μ,λ)(pXY)−λ(μR1+μ¯R2)2+λ(5−μ)=supμ∈[0,1],λ≥0F_(μ,α)(μR1+μ¯R2|pXY).

Step (a) follows from the definition of $F(R_{1},R_{2}|p_{XY})$ and (A16) in Corollary A1. Steps (b) and (c) follow from that

$$\alpha \in \left[0,1/2\right),\lambda =\frac{\alpha }{1-2\alpha }\;\Leftrightarrow \;\lambda \geq 0,\alpha =\frac{\lambda }{1+2\lambda }.$$

From (A19), we have

$$\begin{array}{c} F\left(R_{1},R_{2}|p_{XY}\right)\geq \underset{\mu \in [0,1],\lambda \geq 0}{\sup}{\underline{F}}^{\left(\mu ,\lambda \right)}\left(\mu R_{1}+\bar{\mu }R_{2}|p_{XY}\right)=\underline{F}\left(R_{1},R_{2}|p_{XY}\right), \end{array}$$

completing the proof. □

## Appendix E. Proof of Property 4 Parts c, d, e, and f

In this appendix, we prove Property 4 parts c, d, e, and f. We first prove part c and then prove parts d and e. We finally prove part f.

**Proof** **of** **Property** **4** **part** **c:** We first prove the second inequality in (8) in part c. We first observe that

(A20) $$\begin{array}{c} \exp\left[-{\tilde{\Omega }}^{\left(\mu ,\lambda \right)}\left(p\right)\right]=E_{p}\left[\frac{p_{X}^{\mu \lambda }\left(X\right)p_{Y|U}^{\bar{\mu }\lambda }\left(Y|U\right)}{p_{X|U}^{\mu \lambda }\left(X|U\right)}\right]. \end{array}$$

Let ${\bar{p}}_{X}$ be the uniform distribution on X and let ${\bar{p}}_{Y}$ be the uniform distribution on Y. On lower bound of $\exp[-{\tilde{\Omega }}^{\left(\mu ,\lambda \right)}\left(p\right)]$ for $p\in P_{\mathrm{sh}}\left(p_{XY}\right)$ and $\left(\mu ,\lambda \right)\in {\left[0,1\right]}^{2}$, we have the following chain of inequalities:

(A21) $$\begin{array}{c} \exp\left[-{\tilde{\Omega }}^{\left(\mu ,\lambda \right)}\left(p\right)\right]=\frac{1}{{\left|X\right|}^{\mu \lambda }{\left|Y\right|}^{\bar{\mu }\lambda }}E_{p}\left[\begin{array}{c} \;\\ \;\\ \; \end{array}\right.\;\;p_{X|U}^{-\mu \lambda }\left(X|U\right){\left\{\frac{p_{X}\left(X\right)}{{\bar{p}}_{X}\left(X\right)}\right\}}^{\mu \lambda }{\left\{\frac{p_{Y|U}\left(Y|U\right)}{{\bar{p}}_{Y}\left(Y\right)}\right\}}^{\bar{\mu }\lambda }\left.\begin{array}{c} \;\\ \;\\ \; \end{array}\;\;\right]\\ \overset{\left(a\right)}{\geq }\frac{1}{{\left|X\right|}^{\mu }{\left|Y\right|}^{\bar{\mu }}}E_{p}\left[\begin{array}{c} \;\\ \;\\ \; \end{array}\right.\;\;{\left\{\frac{{\bar{p}}_{X}\left(X\right)}{p_{X}\left(X\right)}\right\}}^{-\mu \lambda }{\left\{\frac{{\bar{p}}_{Y}\left(Y\right)}{p_{Y|U}\left(Y|U\right)}\right\}}^{-\bar{\mu }\lambda }\left.\begin{array}{c} \;\\ \;\\ \; \end{array}\;\;\right]\\ \overset{\left(b\right)}{\geq }\frac{1}{{\left|X\right|}^{\mu }{\left|Y\right|}^{\bar{\mu }}}{\left(E_{p}\left[\begin{array}{c} \;\\ \;\\ \; \end{array}\right.\;\;\frac{{\bar{p}}_{X}\left(X\right)}{p_{X}\left(X\right)}\left.\begin{array}{c} \;\\ \;\\ \; \end{array}\;\;\right]\right)}^{-\mu \lambda }{\left(E_{p}\left[\begin{array}{c} \;\\ \;\\ \; \end{array}\right.\;\;\frac{{\bar{p}}_{Y}\left(Y\right)}{p_{Y|U}\left(Y|U\right)}\left.\begin{array}{c} \;\\ \;\\ \; \end{array}\;\;\right]\right)}^{-\bar{\mu }\lambda }=\frac{1}{{\left|X\right|}^{\mu }{\left|Y\right|}^{\bar{\mu }}}. \end{array}$$

Step (a) follows from that λ∈[0,1] and $p_{X|U}\left(x|u\right)\leq 1$ for any (u,x)∈U×X. Step (b) follows from the reverse Hölder’s inequality. The bound (A21) implies the second inequality in (8). We next show that ${\tilde{\Omega }}^{\left(\mu ,\lambda \right)}\left(p\right)\geq 0$ for λ∈[0,1]. On upper bounds of $\exp[-{\tilde{\Omega }}^{\left(\mu ,\lambda \right)}\left(p\right)]$ for $p\in P_{\mathrm{sh}}\left(p_{XY}\right)$ and λ∈[0,1], we have the following chain of inequalities:

(A22) $$\begin{array}{c} \exp\left[-{\tilde{\Omega }}^{\left(\mu ,\lambda \right)}\left(p\right)\right]\overset{\left(a\right)}{\leq }E_{p}\left[\begin{array}{c} \;\\ \;\\ \; \end{array}\right.\;\;{\left\{\frac{p_{X}\left(X\right)}{p_{X|U}\left(X|U\right)}\right\}}^{\mu \lambda }\left.\begin{array}{c} \;\\ \;\\ \; \end{array}\;\;\right]\overset{\left(b\right)}{\leq }{\left\{E_{p}\left[\begin{array}{c} \;\\ \;\\ \; \end{array}\right.\;\;\frac{p_{X}\left(X\right)}{p_{X|U}\left(X|U\right)}\left.\begin{array}{c} \;\\ \;\\ \; \end{array}\;\;\right]\right\}}^{\mu \lambda }=1. \end{array}$$

Step (a) follows from (A20) and $p_{Y|U}\left(y|u\right)\leq 1$ for any (u,y)∈U×Y. Step (b) follows from μλ∈[0,1] and Hölder’s inequality. □

**Proof** **of** **Property** **4** **parts** **d** **and** **e:** We first prove that, for each $p\in P_{\mathrm{sh}}\left(p_{XY}\right)$ and μ∈[0,1], ${\tilde{\Omega }}^{\left(\mu ,\lambda \right)}\left(p\right)$ is twice differentiable for λ∈[0,1/2]. For simplicity of notations, set

$$\begin{array}{c} \underline{a}:=\left(u,x,y\right),\underline{A}:=\left(U,X,Y\right),\underline{A}:=U\times X\times Y,\\ {\tilde{\omega }}_{p}^{\left(\mu \right)}\left(x,y|u\right):=\varsigma \left(\underline{a}\right),{\tilde{\Omega }}^{\left(\mu ,\lambda \right)}\left(p\right):=\xi \left(\lambda \right). \end{array}$$

Then, we have

(A23) $${\tilde{\Omega }}^{\left(\mu ,\lambda \right)}\left(p\right)=\xi \left(\lambda \right)=-\log\left[\sum_{\underline{a}\in \underline{A}}p_{\underline{A}}\left(\underline{a}\right)e^{-\lambda \varsigma (\underline{a})}\right].$$

The quantity $p^{\left(\lambda \right)}\left(\underline{a}\right)=p_{\underline{A}}^{\left(\lambda \right)}\left(\underline{a}\right),\underline{a}\in A$ has the following form:

(A24) $$p^{\left(\lambda \right)}\left(\underline{a}\right)=e^{\xi (\lambda )}p\left(\underline{a}\right)e^{-\lambda \varsigma (\underline{a})}.$$

By simple computations, we have

(A25) $$\begin{array}{c} \xi^{\prime }\left(\lambda \right)=e^{\xi (\lambda )}\left[\sum_{\underline{a}\in \underline{A}}p\left(\underline{a}\right)\varsigma \left(\underline{a}\right)e^{-\lambda \varsigma (\underline{a})}\right]=\sum_{\underline{a}\in \underline{A}}p^{\left(\lambda \right)}\left(\underline{a}\right)\varsigma \left(\underline{a}\right),\\ \xi^{\prime \prime }\left(\lambda \right)=-e^{2\xi (\lambda )}\left[\sum_{\underline{a},\underline{b}\in \underline{A}}p\left(\underline{a}\right)p_{\underline{A}}\left(\underline{b}\right)\frac{{\left\{\varsigma \left(\underline{a}\right)-\varsigma \left(\underline{b}\right)\right\}}^{2}}{2}e^{-\lambda \left\{\varsigma \left(\underline{a}\right)+\varsigma \left(\underline{b}\right)\right\}}\right]\\ =-\sum_{\underline{a},\underline{b}\in \underline{A}}p^{\left(\lambda \right)}\left(\underline{a}\right)p^{\left(\lambda \right)}\left(\underline{b}\right)\frac{{\left\{\varsigma \left(\underline{a}\right)-\varsigma \left(\underline{b}\right)\right\}}^{2}}{2}=-\sum_{\underline{a}\in \underline{A}}p^{\left(\lambda \right)}\left(\underline{a}\right)\varsigma^{2}\left(\underline{a}\right)+{\left[\sum_{\underline{a}\in \underline{A}}p^{\left(\lambda \right)}\left(\underline{a}\right)\varsigma \left(\underline{a}\right)\right]}^{2}\leq 0. \end{array}$$

On upper bound of $-\xi^{\prime \prime }\left(\lambda \right)\geq 0$ for λ∈[0,1/2], we have the following chain of inequalities:

(A26) $$\begin{array}{c} -\xi^{\prime \prime }\left(\lambda \right)\overset{\left(a\right)}{\leq }\sum_{\underline{a}\in \underline{A}}p^{\left(\lambda \right)}\left(\underline{a}\right)\varsigma^{2}\left(\underline{a}\right)\overset{\left(b\right)}{=}\sum_{\underline{a}\in \underline{A}}p\left(\underline{a}\right)\varsigma^{2}\left(\underline{a}\right)e^{-\lambda \varsigma \left(\underline{a}\right)+\xi \left(\lambda \right)}=e^{\xi (\lambda )}\sum_{\underline{a}\in \underline{A}}p\left(\underline{a}\right)\sqrt{e^{-2\lambda \varsigma (\underline{a})}}\sqrt{\varsigma^{4}\left(\underline{a}\right)}\\ \overset{\left(c\right)}{\leq }\sqrt{e^{2\xi (\lambda )-\xi (2\lambda )}}\sqrt{\sum_{\underline{a}\in \underline{A}}p\left(\underline{a}\right)\varsigma^{4}\left(\underline{a}\right)}\overset{\left(d\right)}{\leq }\sqrt{e^{2\xi (\lambda )}}\sqrt{\sum_{\underline{a}\in \underline{A}}p\left(\underline{a}\right)\varsigma^{4}\left(\underline{a}\right)}. \end{array}$$

Step (a) follows from (A25). Step (b) follows from (A24). Step (c) follows from Cauchy–Schwarz inequality and (A23). Step (d) follows from that ξ(2λ)≥0 for 2λ∈[0,1]. Note that ξ(λ) exists for λ∈[0,1/2]. Furthermore, we have the following:

$$\sum_{\underline{a}\in \underline{A}}p\left(\underline{a}\right)\varsigma^{4}\left(\underline{a}\right)<\infty .$$

Hence, by (A26), $\xi^{\prime \prime }\left(\lambda \right)$ exists for λ∈[0,1/2]. We next prove part e. We derive the lower bound (9) of ${\tilde{\Omega }}^{\left(\mu ,\lambda \right)}\left(p_{XY}\right)$. Fix any (μ,λ)∈[0,1]×[0,1/2] and any $p\in P_{\mathrm{sh}}\left(p_{XY}\right)$. By the Taylor expansion of $\xi \left(\lambda \right)={\tilde{\Omega }}^{\left(\mu ,\lambda \right)}\left(p\right)$ with respect to λ around λ=0, we have that for any $p\in P_{\mathrm{sh}}\left(p_{XY}\right)$ and for some ν∈[0,λ]

(A27) $$\begin{array}{c} {\tilde{\Omega }}^{\left(\mu ,\lambda \right)}\left(p\right)=\xi \left(0\right)+\xi^{\prime }\left(0\right)\lambda +\frac{1}{2}\xi^{\prime \prime }\left(\nu \right)\lambda^{2}=\lambda E_{p}\left[{\tilde{\omega }}_{p}^{\left(\mu \right)}\left(X,Y|U\right)\right]-\frac{\lambda^{2}}{2}{\mathrm{Var}}_{p^{\left(\nu \right)}}\left[{\tilde{\omega }}_{p}^{\left(\mu \right)}\left(X,Y|U\right)\right]\\ \overset{\left(a\right)}{\geq }\lambda R^{\left(\mu \right)}\left(p_{XY}\right)-\frac{\lambda^{2}}{2}{\mathrm{Var}}_{p^{\left(\nu \right)}}\left[{\tilde{\omega }}_{p}^{\left(\mu \right)}\left(X,Y,Z|U\right)\right]. \end{array}$$

Step (a) follows from $p\in P_{\mathrm{sh}}\left(p_{XY}\right)$,

$$E_{p}\left[{\tilde{\omega }}_{p}^{\left(\mu \right)}\left(X,Y|U\right)\right]=\mu I_{p}\left(X;U\right)+\bar{\mu }H_{p}\left(Y|U\right),$$

and the definition of $R^{\left(\mu \right)}\left(p_{XY}\right)$. Let $\left(\nu_{\mathrm{opt}},p_{\mathrm{opt}}\right)$ ∈[0,λ]×$P_{\mathrm{sh}}\left(p_{XY}\right)$ be a pair which attains $\rho^{\left(\mu ,\lambda \right)}\left(p_{XY}\right)$. By this definition, we have that

(A28) $$\begin{array}{c} {\tilde{\Omega }}^{\left(\mu ,\lambda \right)}\left(p_{\mathrm{opt}}\right)={\tilde{\Omega }}^{\left(\mu ,\lambda \right)}\left(p_{XY}\right) \end{array}$$

and that, for any ν∈[0,λ],

(A29) $$\begin{array}{c} {\mathrm{Var}}_{p_{\mathrm{opt}}^{\left(\nu \right)}}\left[\omega_{p_{\mathrm{opt}}}^{\left(\mu \right)}\left(X,Y|U\right)\right]\leq {\mathrm{Var}}_{p_{\mathrm{opt}}^{\left(\nu_{\mathrm{opt}}\right)}}\left[\omega_{p_{\mathrm{opt}}}^{\left(\mu \right)}\left(X,Y|U\right)\right]=\rho^{\left(\mu ,\lambda \right)}\left(p_{XY}\right). \end{array}$$

On lower bounds of $\Omega^{\left(\mu ,\lambda \right)}\left(p_{XY}\right)$, we have the following chain of inequalities:

$$\begin{array}{c} {\tilde{\Omega }}^{\left(\mu ,\lambda \right)}\left(p_{XY}\right)\overset{\left(a\right)}{=}{\tilde{\Omega }}^{\left(\mu ,\lambda \right)}\left(p_{\mathrm{opt}}\right)\overset{\left(b\right)}{\geq }\lambda R^{\left(\mu \right)}\left(p_{XY}\right)-\frac{\lambda^{2}}{2}{\mathrm{Var}}_{p_{\mathrm{opt}}^{\left(\nu \right)}}\left[{\tilde{\omega }}_{p_{\mathrm{opt}}}^{\left(\mu \right)}\left(X,Y|U\right)\right]\\ \overset{\left(c\right)}{\geq }\lambda R^{\left(\mu \right)}\left(p_{XY}\right)-\frac{\lambda^{2}}{2}\rho^{\left(\mu ,\lambda \right)}\left(p_{XY}\right)\overset{\left(d\right)}{\geq }\lambda R^{\left(\mu \right)}\left(p_{XY}\right)-\frac{\lambda^{2}}{2}\rho \left(p_{XY}\right). \end{array}$$

Step (a) follows from (A28). Step (b) follows from (A27). Step (c) follows from (A29). Step (d) follows from the definition of $\rho (p_{XY})$. □

To prove part f, we use the following lemma.

**Lemma** **A6.**
*When τ∈(0,(1/2)ρ], the maximum of*

$$\frac{1}{2+5\lambda }\left\{-\frac{\rho }{2}\lambda^{2}+\tau \lambda \right\}$$

*for λ∈(0,1/2] is attained by the positive $\lambda_{0}$ satisfying*

(A30) $$\vartheta \left(\lambda_{0}\right):=\lambda_{0}+\frac{5}{4}\lambda_{0}^{2}=\frac{\tau }{\rho }.$$

*Let g(a) be the inverse function of ϑ(a) for a≥0. Then, the condition of (A30) is equivalent to $\lambda_{0}=g\left(\frac{\tau }{\rho }\right)$. The maximum is given by*

$$\frac{1}{2+5\lambda_{0}}\left\{-\frac{\rho }{2}\lambda_{0}^{2}+\tau \lambda_{0}\right\}=\frac{\rho }{4}\lambda_{0}^{2}=\frac{\rho }{4}g^{2}\left(\frac{\tau }{\rho }\right).$$

By an elementary computation, we can prove this lemma. We omit the detail.

**Proof of Property 4 part** **f.** By the hyperplane expression $R_{\mathrm{sh}}\left(p_{XY}\right)$ of $R(p_{XY})$ stated Property 3 part b, we have that, when $\left(R_{1}+\tau ,R_{2}+\tau \right)\notin R\left(p_{XY}\right)$, we have

(A31) $$\begin{array}{c} R^{\left(\mu_{0}\right)}\left(p_{XY}\right)-\left(\mu_{0}R_{1}+\bar{\mu_{0}}R_{2}\right)>\tau \end{array}$$

for some $\mu_{0}\in \left[0,1\right]$. Then, for each positive τ, we have the following chain of inequalities:

$$\begin{array}{c} \underline{F}\left(R_{1},R_{2}|p_{XY}\right)\geq \underset{\lambda \in (0,1/2]}{\sup}{\underline{F}}^{\left(\mu_{0},\lambda \right)}\left(\mu_{0}R_{1}+\bar{\mu_{0}}R_{2}|p_{XY}\right)=\underset{\lambda \in (0,1/2]}{\sup}\frac{{\tilde{\Omega }}^{\left(\mu_{0},\lambda \right)}\left(p_{XY}\right)-\lambda \left(\mu_{0}R_{1}+\bar{\mu_{0}}R_{2}\right)}{2+\lambda (5-\mu_{0})}\\ \overset{\left(a\right)}{\geq }\underset{\lambda \in (0,1/2]}{\sup}\frac{1}{2+5\lambda }\left\{-\frac{\rho }{2}\lambda^{2}\right.+\lambda R^{\left(\mu_{0}\right)}\left(p_{XY}\right)-\lambda \left(\mu_{0}R_{1}+\bar{\mu_{0}}R_{2}\right)\}\\ \overset{\left(b\right)}{>}\underset{\lambda \in (0,1/2]}{\sup}\frac{1}{2+5\lambda }\left\{-\frac{\rho }{2}\lambda^{2}+\tau \lambda \right\}\overset{\left(c\right)}{=}\frac{\rho }{4}g^{2}\left(\frac{\tau }{\rho }\right). \end{array}$$

Step (a) follows from Property 4 part d. Step (b) follows from (A31). Step (c) follows from Lemma A6. □

## Appendix F. Proof of Lemma 1

To prove Lemma 1, we prepare a lemma. Set

$$A_{n}:=\left\{\left(s,x^{n},y^{n}\right):\frac{1}{n}\log\frac{p_{SX^{n}Y^{n}}\left(s,x^{n},y^{n}\right)}{{\hat{q}}_{SX^{n}Y^{n}}\left(s,x^{n},y^{n}\right)}\geq -\eta \right\}.$$

Furthermore, set

$$\begin{array}{c} {\tilde{B}}_{n}:=\left\{x^{n}:\frac{1}{n}\log\frac{p_{X^{n}}\left(x^{n}\right)}{Q_{X^{n}}\left(x^{n}\right)}\geq -\eta \right\},B_{n}:={\tilde{B}}_{n}\times M_{1}\times Y^{n},B_{n}^{c}:={\tilde{B}}_{n}^{c}\times M_{1}\times Y^{n},\\ {\tilde{C}}_{n}:=\{\left(s,x^{n}\right):\begin{array}{c} s=\varphi_{1}^{\left(n\right)}\left(x^{n}\right),{\tilde{Q}}_{X^{n}|S}\left(x^{n}|s\right)\leq M_{1}e^{n\eta }p_{X^{n}}\left(x^{n}\right)\}, \end{array}C_{n}:={\tilde{C}}_{n}\times Y^{n},C_{n}^{c}:={\tilde{C}}_{n}^{c}\times Y^{n},\\ D_{n}:=\{\left(s,x^{n},y^{n}\right):\begin{array}{c} s=\varphi_{1}^{\left(n\right)}\left(x^{n}\right),p_{Y^{n}|S}\left(y^{n}|s\right)\geq \left(1/M_{2}\right)e^{-n\eta }\}, \end{array}\\ E_{n}:=\{\left(s,x^{n},y^{n}\right):\begin{array}{c} s=\varphi_{1}^{\left(n\right)}\left(x^{n}\right),\psi^{\left(n\right)}\left(\varphi_{1}^{\left(n\right)}\left(x^{n}\right),\varphi_{2}^{\left(n\right)}\left(y^{n}\right)\right)=y^{n}\}. \end{array} \end{array}$$

Then, we have the following lemma.

**Lemma** **A7.**

$$\begin{array}{c} p_{SX^{n}Y^{n}}\left(A_{n}^{c}\right)\leq e^{-n\eta },p_{SX^{n}Y^{n}}\left(B_{n}^{c}\right)\leq e^{-n\eta },p_{SX^{n}Y^{n}}\left(C_{n}^{c}\right)\leq e^{-n\eta },p_{SX^{n}Y^{n}}\left(D_{n}^{c}\cap E_{n}\right)\leq e^{-n\eta }. \end{array}$$

**Proof.** We first prove the first inequality.

$$\begin{array}{c} p_{SX^{n}Y^{n}}\left(A_{n}^{c}\right)=\sum_{\left(s,x^{n},y^{n}\right)\in A_{n}^{c}}p_{SX^{n}Y^{n}}\left(s,x^{n},y^{n}\right)\\ \overset{\left(a\right)}{\leq }\sum_{\left(s,x^{n},y^{n}\right)\in A_{n}^{c}}e^{-n\eta }{\hat{q}}_{SX^{n}Y^{n}}\left(s,x^{n},y^{n}\right)=e^{-n\eta }{\hat{q}}_{SX^{n}Y^{n}}\left(A_{n}^{c}\right)\leq e^{-n\eta }. \end{array}$$

Step (a) follows from the definition of $A_{n}$. In the second inequality, we have

$$\begin{array}{c} p_{SX^{n}Y^{n}}\left(B_{n}^{c}\right)=p_{X^{n}}\left({\tilde{B}}_{n}^{c}\right)=\sum_{x^{n}\in {\tilde{B}}_{n}^{c}}p_{X_{n}}\left(x^{n}\right)\overset{\left(a\right)}{\leq }\sum_{x^{n}\in {\tilde{B}}_{n}^{c}}e^{-n\eta }Q_{X^{n}}\left(x^{n}\right)=e^{-n\eta }Q_{X^{n}}\left({\tilde{B}}_{n}^{c}\right)\leq e^{-n\eta }. \end{array}$$

Step (a) follows from the definition of $B_{n}$. We next prove the third inequality:

pSXnYn(Cnc)=pSXn(C˜nc)=∑s∈M1∑xn:φ1(n)(xn)=spXn(xn)≤(1/M1)e−nη×Q˜Xn|S(xn|s)pXn(xn)≤1M1e−nη∑s∈M1∑xn:φ1(n)(xn)=spXn(xn)≤(1/M1)e−nη×Q˜Xn|S(xn|s)Q˜Xn|S(xn|s)≤1M1e−nη|M1|=e−nη.

Finally, we prove the fourth inequality. We first observe that

$$\begin{array}{c} p_{S}\left(s\right)=\sum_{x^{n}:\varphi_{1}^{\left(n\right)}\left(x^{n}\right)=s}p_{X^{n}}\left(x^{n}\right),\;p_{X^{n}|S}\left(x^{n}|s\right)=\frac{p_{X^{n}}\left(x^{n}\right)}{p_{S}\left(s\right)}. \end{array}$$

We have the following chain of inequalities:

pSXnYnDnc∩En=∑s∈M1pS(s)∑xn:φ1(n)(xn)=spXn|S(xn|s)∑yn:ψ(n)(s,φ2(n)(yn))=ynpYn|S(yn|s)≤(1/M2)e−nηpYn|Xn(yn|xn)=∑s∈M1pS(s)∑yn:ψ(n)(s,φ2(n)(yn))=ynpYn|S(yn|s)≤(1/M2)e−nηpYn|S(yn|s)≤∑s∈M1pS(s)1M2e−nηyn:ψ(n)(s,φ2(n)(yn))=yn≤(a)∑s∈M1pS(s)1M2e−nηM2=e−nη.

Step (a) follows from that the number of $y^{n}$ correctly decoded does not exceed $M_{2}$. □

**Proof** **of** **Lemma** **1:** By definition, we have

$$\begin{array}{cc} p_{SX^{n}Y^{n}}\left(A_{n}\cap B_{n}\cap C_{n}\cap D_{n}\right)=p_{SX^{n}Y^{n}} & \left\{\frac{1}{n}\log\frac{p_{SX^{n}Y^{n}}\left(S,X^{n},Y^{n}\right)}{{\hat{q}}_{{SX^{n}Y}^{n}}\left(S,X^{n},Y^{n}\right)}\geq -\eta ,\right.\\ & \;0\geq \frac{1}{n}\log\frac{Q_{X^{n}}\left(X^{n}\right)}{p_{X^{n}}\left(X^{n}\right)}-\eta ,\\ & \;\;\frac{1}{n}\log M_{1}\geq \frac{1}{n}\log\frac{{\tilde{Q}}_{X^{n}|S}\left(X^{n}|S\right)}{p_{X^{n}}\left(X^{n}\right)}-\eta ,\\ & \;\;\frac{1}{n}\log M_{2}\geq \left.\frac{1}{n}\log\frac{1}{p_{Y^{n}|S}\left(Y^{n}|S\right)}-\eta \right\}. \end{array}$$

Then, for any $(\varphi_{1}^{\left(n\right)}$, $\varphi_{2}^{\left(n\right)},\psi^{\left(n\right)})$ satisfying $\left(1/n\right)\log\left|\right|\varphi_{i}^{\left(n\right)}\left|\right|\leq R_{i},i=1,2,$ we have

$$\begin{array}{cc} p_{SX^{n}Y^{n}}\left(A_{n}\cap B_{n}\cap C_{n}\cap D_{n}\right)\leq p_{SX^{n}Y^{n}} & \left\{\frac{1}{n}\log\frac{p_{SX^{n}Y^{n}}\left(S,X^{n},Y^{n}\right)}{{\hat{q}}_{{SX^{n}Y}^{n}}\left(S,X^{n},Y^{n}\right)}\geq -\eta ,\right.\\ & \;0\geq \frac{1}{n}\log\frac{Q_{X^{n}}\left(X^{n}\right)}{p_{X^{n}}\left(X^{n}\right)}-\eta ,\\ & \;\;R_{1}\geq \frac{1}{n}\log\frac{{\tilde{Q}}_{X^{n}|S}\left(X^{n}|S\right)}{p_{X^{n}}\left(X^{n}\right)}-\eta ,\\ & \;\;R_{2}\geq \left.\frac{1}{n}\log\frac{1}{p_{Y^{n}|S}\left(Y^{n}|S\right)}-\eta \right\}. \end{array}$$

Hence, it suffices to show

$$\begin{array}{cc} P_{c}^{\left(n\right)}\left(\varphi_{1}^{\left(n\right)},\varphi_{2}^{\left(n\right)},\psi^{\left(n\right)}\right) & \leq p_{SX^{n}Y^{n}}\left(A_{n}\cap B_{n}\cap C_{n}\cap D_{n}\right)+4e^{-n\eta } \end{array}$$

to prove Lemma 1. By definition, we have $P_{c}^{\left(n\right)}\left(\varphi_{1}^{\left(n\right)},\varphi_{2}^{\left(n\right)},\psi^{\left(n\right)}\right)=p_{SX^{n}Y^{n}}\left(E_{n}\right).$ Then, we have the following.

$$\begin{array}{c} P_{c}^{\left(n\right)}\left(\varphi_{1}^{\left(n\right)},\varphi_{2}^{\left(n\right)},\psi^{\left(n\right)}\right)=p_{SX^{n}Y^{n}}\left(E_{n}\right)\\ =p_{SX^{n}Y^{n}}\left(A_{n}\cap B_{n}\cap C_{n}\cap D_{n}\cap E_{n}\right)+p_{SX^{n}Y^{n}}\left({\left[A_{n}\cap B_{n}\cap C_{n}\cap D_{n}\right]}^{c}\cap E_{n}\right)\\ \leq p_{SX^{n}Y^{n}}\left(A_{n}\cap B_{n}\cap C_{n}\cap D_{n}\right)+p_{SX^{n}Y^{n}}\left(A_{n}^{c}\right)+p_{SX^{n}Y^{n}}\left(B_{n}^{c}\right)+p_{SX^{n}Y^{n}}\left(C_{n}^{c}\right)+p_{SX^{n}Y^{n}}\left(D_{n}^{c}\cap E_{n}\right)\\ \overset{\left(a\right)}{\leq }p_{SX^{n}Y^{n}}\left(A_{n}\cap B_{n}\cap C_{n}\cap D_{n}\right)+4e^{-n\eta }. \end{array}$$

Step (a) follows from Lemma A7. □

## Appendix G. Proof of Lemma 3

In this appendix, we prove Lemma 3.

**Proof** **of** **Lemma** **3:** We first prove the Markov chain $SX^{t-1}↔X_{t}↔Y_{t}$ in (18) in Lemma 3. We have the following chain of inequalities:

$$\begin{array}{c} I\left(Y_{t};SX^{t-1}|X_{t}\right)=H\left(Y_{t}|X_{t}\right)-H\left(Y_{t}|SX^{t-1}X_{t}\right)\leq H\left(Y_{t}|X_{t}\right)-H\left(Y_{t}|SX^{n}\right)\\ \overset{\left(a\right)}{=}H\left(Y_{t}|X_{t}\right)-H\left(Y_{t}|X^{n}\right)\overset{\left(b\right)}{=}H\left(Y_{t}|X_{t}\right)-H\left(Y_{t}|X_{t}\right)=0. \end{array}$$

Step (a) follows from that $S=\varphi_{1}^{\left(n\right)}\left(X^{n}\right)$ is a function of $X^{n}$. Step (b) follows from the memoryless property of the information source ${\left\{\left(X_{t},Y_{t}\right)\right\}}_{t=1}^{\infty }$. Next, we prove the Markov chain $Y^{t-1}↔SX^{t-1}↔\left(X_{t},Y_{t}\right)$ in (19) in Lemma 3. We have the following chain of inequalities:

$$\begin{array}{c} I\left(X_{t}Y_{t};Y^{t-1}|SX^{t-1}\right)=H\left(Y^{t-1}|SX^{t-1}\right)-H\left(Y^{t-1}|SX^{t-1}X_{t}Y_{t}\right)\leq H\left(Y^{t-1}|X^{t-1}\right)-H\left(Y^{t-1}|X^{n}SY_{t}\right)\\ \overset{\left(a\right)}{=}H\left(Y^{t-1}|X^{t-1}\right)-H\left(Y^{t-1}|X^{n}Y_{t}\right)\overset{\left(b\right)}{=}H\left(Y^{t-1}|X^{t-1}\right)-H\left(Y^{t-1}|X^{t-1}Y_{t}\right)=0. \end{array}$$

Step (a) follows from that $S=\varphi_{1}^{\left(n\right)}\left(X^{n}\right)$ is a function of $X^{n}$. Step (b) follows from the memoryless property of the information source ${\left\{\left(X_{t},Y_{t}\right)\right\}}_{t=1}^{\infty }$. □

## Appendix H. Proof of Lemma 6

In this appendix, we prove Lemma 6.

**Proof** **of** **Lemma** **6.** By the definition of $p_{SX^{t}Y^{t};F^{t}}^{\left(\mu ,\alpha \right)}(s,$$x^{t},y^{t})$, for t=1,2,⋯,n, we have

(A32) $$\begin{array}{c} p_{SX^{t}Y^{t};F^{t}}^{\left(\mu ,\alpha \right)}\left(s,x^{t},y^{t}\right)=C_{t}^{-1}p_{SX^{t}Y^{t}}\left(s,x^{t},y^{t}\right)\prod_{i=1}^{t}f_{F_{i}}^{\left(\mu ,\alpha \right)}\left(x_{i},y_{i}|u_{i}\right). \end{array}$$

Then, we have the following chain of equalities:

(A33) $$\begin{array}{c} p_{SX^{t}Y^{t};F^{t}}^{\left(\mu ,\alpha \right)}\left(s,x^{t},y^{t}\right)\overset{\left(a\right)}{=}C_{t}^{-1}p_{SX^{t}Y^{t}}\left(s,x^{t},y^{t}\right)\prod_{i=1}^{t}f_{F_{i}}^{\left(\mu ,\alpha \right)}\left(x_{i},y_{i}|u_{i}\right)\\ =C_{t}^{-1}p_{SX^{t-1}Y^{t-1}}\left(s,x^{t-1},y^{t-1}\right)\prod_{i=1}^{t-1}f_{F_{i}}^{\left(\mu ,\alpha \right)}\left(x_{i},y_{i}|u_{i}\right)\\ \;\times p_{X_{t}Y_{t}|SX^{t-1}Y^{t-1}}\left(x_{t},y_{t}|s,x^{t-1},y^{t-1}\right)f_{F_{t}}^{\left(\mu ,\alpha \right)}\left(x_{t},y_{t}|u_{t}\right)\\ \overset{\left(b\right)}{=}C_{t}^{-1}C_{t-1}p_{SX^{t-1}Y^{t-1}}^{\left(\mu ,\alpha \right)}\left(s,x^{t-1},y^{t-1}\right)p_{X_{t}Y_{t}|SX^{t-1}Y^{t-1}}\left(x_{t},y_{t}|s,x^{t-1},y^{t-1}\right)f_{F_{t}}^{\left(\mu ,\alpha \right)}\left(x_{t},y_{t}|u_{t}\right)\\ ={\left(\Phi_{t}^{\left(\mu ,\alpha \right)}\right)}^{-1}p_{SX^{t-1}Y^{t-1};F^{t-1}}^{\left(\mu ,\alpha \right)}\left(s,x^{t-1},y^{t-1}\right)p_{X_{t}Y_{t}|SX^{t-1}Y^{t-1}}\left(x_{t},y_{t}|s,x^{t-1},y^{t-1}\right)f_{F_{t}}^{\left(\mu ,\alpha \right)}\left(x_{t},y_{t}|u_{t}\right). \end{array}$$

Steps (a) and (b) follow from (A32). From (A33), we have

(A34) $$\begin{array}{c} \Phi_{t}^{\left(\mu ,\alpha \right)}p_{SX^{t}Y^{t};F^{t}}^{\left(\mu ,\alpha \right)}\left(s,x^{t},y^{t}\right) \end{array}$$

(A35) $$\begin{array}{c} =p_{SX^{t-1}Y^{t-1};F^{t-1}}^{\left(\mu ,\alpha \right)}\left(s,x^{t-1},y^{t-1}\right)p_{X_{t}Y_{t}|SX^{t-1}Y^{t-1}}\left(x_{t},y_{t}|s,x^{t-1},y^{t-1}\right)f_{F_{t}}^{\left(\mu ,\alpha \right)}\left(x_{t},y_{t}|u_{t}\right). \end{array}$$

Taking summations of (A34) and (A35) with respect to $s,x^{t},y^{t}$, we obtain

$$\begin{array}{c} \Phi_{t}^{\left(\mu ,\alpha \right)}=\sum_{s,x^{t},y^{t}}p_{SX^{t-1}Y^{t-1};F^{t-1}}^{\left(\mu ,\alpha \right)}\left(s,x^{t-1},y^{t-1}\right)p_{X_{t}Y_{t}|SX^{t-1}Y^{t-1}}\left(x_{t},y_{t}|s,x^{t-1},y^{t-1}\right)f_{F_{t}}^{\left(\mu ,\alpha \right)}\left(x_{t},y_{t}|u_{t}\right), \end{array}$$

completing the proof. □

## References

[1] Ahlswede R.F., Körner J. (1975). Source coding with side information and a converse for degraded broadcast channels. IEEE Trans. Inf. Theory.

[2] Wyner A.D. (1975). On source coding with side information at the decoder. IEEE Trans. Inf. Theory.

[3] Csiszár I., Longo G. (1971). On the exponent function for source coding and for testing simple statistical hypotheses. Studia Sci. Math. Hungar.

[4] Slepian D., Wolf J.K. (1973). Noiseless coding of correlated information sources. IEEE Trans. Inf. Theory.

[5] Oohama Y., Han T.S. (1994). Universal coding for the Slepian-wolf data compression system and the strong converse theorem. IEEE Trans. Inf. Theory.

[6] Ahlswede R., Gács P., Körner J. (1976). Bounds on conditional probabilities with applications in multi-user communication. Probab. Theory Relat. Fields.

[7] Gu W., Effors M. A strong converse for a collection of network source coding problems. Proceedings of the IEEE International Symposium on Information Theory.

[8] Oohama Y. Strong converse exponent for degraded broadcast channels at rates outside the capacity region. Proceedings of the 2015 IEEE International Symposium on Information Theory.

[9] Oohama Y. Strong converse theorems for degraded broadcast channels with feedback. Proceedings of the 2015 IEEE International Symposium on Information Theory.

[10] Oohama Y. Exponent function for asymmetric broadcast channels at rates outside the capacity region. Proceedings of the 2016 IEEE International Symposium on Information Theory and its Applications.

[11] Oohama Y. New Strong Converse for Asymmetric Broadcast Channels. https://arxiv.org/pdf/1604.02901.pdf.

[12] Oohama Y. (2018). Exponential strong converse for source coding with side information at the decoder. Entropy.

[13] Watanabe S. A converse bound on Wyner-Ahlswede-Körner network via Gray–Wyner network. Proceedings of the 2017 IEEE Information Theory Workshop (ITW).

[14] Liu J., van Handel R., Verdu S. Beyond the blowing-up lemma: Sharp converses via reverse hypercontractivity. Proceedings of the 2017 IEEE International Symposium on Information Theory (ISIT).

[15] Watanabe S. (2017). Second-order region for Gray–Wyner network. IEEE Trans. Inform. Theory.

[16] Liu J. Dispersion bound for the Wyner-Ahlswede-Körner network via reverse hypercontractivity on types. Proceedings of the 2018 IEEE International Symposium on Information Theory (ISIT).

[17] Watanabe S., Oohama Y. Privacy amplification theorem for bounded storage eavesdropper. Proceedings of the 2012 IEEE Information Theory Workshop (ITW).

[18] Oohama Y., Santoso B. Information Theoretic Security for Side-Channel Attacks to the Shannon Cipher System. https://arxiv.org/pdf/1801.02563v5.pdf..

[19] Santoso B., Oohama Y. (2019). Information Theoretic Security for Shannon Cipher System under Side-Channel Attacks. Entropy.

[20] Oohama Y. (2015). Exponent Function for One Helper Source Coding Problem at Rates outside the Rate Region. arXiv.

[21] Csiszár I., Körner J. (1981). Information Theory: Coding Theorems for Discrete Memoryless Systems.

[22] Han T.S. (2002). Information-Spectrum Methods in Information Theory.

